# Bioengineer mesenchymal stem cell for treatment of glioma by IL‐12 mediated microenvironment reprogramming and nCD47‐SLAMF7 mediated phagocytosis regulation of macrophages

**DOI:** 10.1002/EXP.20240027

**Published:** 2024-06-25

**Authors:** Man Li, Lisen Lu, Qungen Xiao, Ali Abdi Maalim, Bin Nie, Yanchao Liu, Ulf D. Kahlert, Kai Shu, Ting Lei, Mingxin Zhu

**Affiliations:** ^1^ Department of Anesthesiology and Pain Medicine Hubei Key Laboratory of Geriatric Anesthesia and Perioperative Brain Health and Wuhan Clinical Research Center for Geriatric Anesthesia Tongji Hospital Tongji Medical College Huazhong University of Science and Technology Wuhan People's Republic of China; ^2^ Department of Neurosurgery Tongji Hospital Tongji Medical College Huazhong University of Science and Technology Wuhan People's Republic of China; ^3^ College of Biomedicine and Health and College of Life Science and Technology Huazhong Agricultural University Wuhan China; ^4^ Molecular and Experimental Surgery Clinic for General‐, Visceral‐, Vascular and Transplant Surgery Faculty of Medicine and University Hospital Magdeburg Otto‐von‐Guericke University Magdeburg Germany

**Keywords:** CD47‐SLAMF7, glioma, mesenchymal stem cells

## Abstract

High expression of cellular self‐activated immunosuppressive molecules and extensive infiltration of suppressive immune cells in the tumor microenvironment are the main factors contributing to glioma's resistance to immunotherapy. Nonetheless, technology to modify the expression of glioma cellular self‐molecules through gene editing requires further development. This project advances cell therapy strategies to reverse the immunosuppressive microenvironment of glioma (TIME). Bone marrow‐derived mesenchymal stem cells (MSCs) are engineered to express bioactive proteins and demonstrate tumor‐homing characteristics upon activation by TGF‐β. These MSCs are designed to secrete the anti‐tumor immune cytokine IL‐12 and the nCD47‐SLAMF7 fusion protein, which regulates T‐cell activity and macrophage phagocytosis. The engineered MSCs are then injected in situ into the glioma site, circumventing the blood‐brain barrier to deliver high local concentrations of bioactive proteins. This approach aims to enhance the M1 polarization of infiltrating macrophages, stimulate macrophage‐mediated tumor cell phagocytosis, activate antigen‐presenting cells, and promote effector CD8^+^ T cell infiltration, effectively controlling glioma. Additionally, the engineered MSCs may serve as a universal treatment for other tumors that express TGF‐β at high levels. This study proposes a novel treatment strategy for the clinical management of glioma patients.

## Graphical abstract

High expression of cellular self‐activated immunosuppressive molecules and extensive infiltration of suppressive immune cells in the tumor microenvironment are the main factors leading to the resistance of glioma to immunotherapy. This study constructed engineering MSCs expressing IL‐12 and nCD47‐SLAMF7 (anti‐CD47 nanobody combined with the extracellular domain of SLAMF7). Intracranial injection of these two kinds of engineered MSCs could promote M1 polarization of infiltrating macrophages, phagocytosis of tumor cells by macrophages and antigen‐presenting cells, and activation of effector T cells. In addition, the two kinds of engineered MSCs could also promote the release of a large number of pro‐inflammatory cytokines in the tumor microenvironment, reverse the tumor‐inhibitory immune microenvironment, and thus inhibit the development of glioma cells.

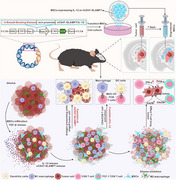

## 1 INTRODUCTION

Gliomas are the most common primary tumor of the central nervous system.^[^ ^1^ ^]^ The tumor's unique location and its invasive growth characteristics make complete removal of glioma challenging. Furthermore, the existence of the blood‐brain barrier and the interaction between neurons and the tumor limit the effect of clinical radiotherapy and chemotherapy.^[^ ^2^ ^]^ Consequently, the average survival time of glioma patients is only 14 months. In recent years, immune checkpoint blocking (ICB) has achieved a breakthrough and has been successfully used in the treatment of many types of tumors, but its therapeutic effect on glioma is not ideal.^[^ ^3^ ^]^ The primary reason for this is the immunosuppressive environment within the tumor, which arises due to extensive infiltration by myeloid‐derived suppressor cells (MDSCs), the enhanced differentiation of tumor‐associated macrophages (TAMs), and the abnormal proliferation of blood vessels.^[^ ^4^ ^]^

The factors contributing to glioma's resistance to immunotherapy include both endogenous and exogenous elements. Endogenous factors involve the activation of the β‐catenin signaling pathway,^[^ ^5^ ^]^ high expression of PD‐L1,^[^ ^6^ ^]^ and high expression of immune inhibitory molecules, such as IDO^[^ ^7^ ^]^ and PEG2.^[^ ^8^ ^]^ However, there are significant technical challenges in modifying the gene expression profile of glioma cells in vivo. On the other hand, exogenous factors include the infiltration of various immunosuppressive cells and the expression of related inhibitory molecules,^[^ ^9^ ^]^ while macrophages constitute more than half of the immune cells infiltrating glioma, secreting immunosuppressive molecules like IL‐4, IL‐10, and SIRRα.^[^ ^10^ ^]^ These molecules can effectively enable glioma cells to evade innate immune recognition, thus limiting the efficacy of adaptive immune responses. Moreover, the interaction between CD47 and SIRPα has been recognized as a classical immunosuppressive signaling pathway.^[^ ^11^ ^]^ Radiotherapy has been found to increase the expression of CD47 in glioma patients by altering mitochondrial fatty acid oxidation levels.^[^ ^12^ ^]^ The trans‐interactions of the CD47/SIRPα axis and cis‐interaction of CD47/SLAMF7 (member of the signaling lymphocyte activating molecule family 7) inhibits phagocytosis and antigen presentation by phagocyte‐like cells to tumor cells, which in turn inhibits the killing effect of innate immune cells on tumor cells and further inhibits direct recognition by adaptive immune cells.^[^ ^13^ ^]^ However, since CD47 is also expressed by blood cells and other normal cells, the use of anti‐CD47 antibody therapy is limited in its effectiveness.^[^ ^14^ ^]^ As an important indicator of the regulation of tumor phagocytosis by phagocytic cells, SLAMF7 has been employed in the treatment of triple‐negative breast cancer using a bispecific nanobioconjugate, demonstrating promising efficacy.^[^ ^15^ ^]^ Therefore, developing a strategy to locally modulate CD47 activity and inhibit its interaction with SLAMF7 could effectively counteract the contribution of exogenous factors to immunotherapy resistance in glioblastoma multiforme (GBM).

Cell therapy plays a crucial role in anti‐tumor immunity, including CAR‐T cells, TCR‐T cells, CAR‐macrophages, and CAR‐NK cells, all of which have demonstrated tumor‐killing efficacy.^[^ ^16^ ^]^ However, its application is limited due to MHC restriction, tumor cell heterogeneity, and the presence of an immune‐regulatory microenvironment.^[^ ^17^ ^]^ Mesenchymal stem cells (MSCs) are a group of cells characterized by a low degree of differentiation, strong proliferative capacity, high differentiation potential, and a unique ability to “home” to tumors.^[^ ^18^ ^]^ MSCs have been used for preclinical GBM therapy in a variety of forms, including MSCs themselves,^[^ ^19^ ^]^ MSC‐derived extracellular vesicles,^[^ ^20^ ^]^ oncolytic virus vectors,^[^ ^21^ ^]^ suicide protein expression containers,^[^ ^22^ ^]^ and immune‐activating cytokine vectors.^[^ ^23^ ^]^ Previous work in this study developed a promoter system that is responsive to TGF‐β, using the properties of MSCs to react to this cytokine. This system specifically enhanced the expression of relevant cytokines by MSCs within the GBM microenvironment, which is rich in TGF‐β. This, in turn, significantly inhibited the growth of a mouse glioma model.^[^ ^24^ ^]^ This project continues to capitalize on the advantages of MSCs in anti‐tumor immunotherapy and achieves the reversal of the glioma immunosuppressive microenvironment (TIME) through genetic programming, effectively inhibiting the in vivo growth of GL261 tumor cells.

As IL‐12 cytokines have been reported to enhance natural and cellular immune effects in a variety of tumors,^[^ ^25^ ^]^ MSCs were designed to express IL‐12 cytokines and nCD47‐SLAMF7 fusion protein under the control of TGF‐β concentration. NCD47‐SLAMF7 consists of a signaling peptide, a nanobody that can block the function of CD47, and the extracellular region of SLAMF7, which can target tumor cells and mask their CD47 molecules. This allows tumor cells to present SLAMF7 on their surface. The strategy facilitates the uptake of tumor cells by SLAMF7‐expressing macrophages and activates innate and cellular immunity in glioma, thereby effectively controlling the progression of the disease. In this study, we found that the levels of IL‐12 and SLAMF7 expressed by clinical tumor patients were not sufficient to regulate the tumor immunosuppressive microenvironment (TIME), as determined through analysis of clinical databases and the Human Protein Atlas. Furthermore, the CD47/SIRPα axis expression in the mouse glioma model was consistent with that in clinical patients. Genetically engineered MSCs, with their homing properties, have been demonstrated to effectively release IL‐12 cytokines and the nCD47‐SLAMF7 fusion protein by activating the Smad4 transcription factor in a TGF‐β‐rich environment. Additionally, supernatants from MSCs expressing these proteins were found to effectively promote T cell proliferation and activation, as well as enhance the phagocytic and antigen‐presenting abilities of macrophages against tumor cells. The results of in vitro and in vivo experiments demonstrated that MSCs expressing the aforementioned proteins could effectively home to tumor sites. These MSCs were shown to promote macrophage‐mediated phagocytosis of tumor cells and the activation of effector T cells, as well as the in vivo release of cytokines associated with anti‐tumor immunity. Consequently, this led to the effective containment of glioma model development and provides a new therapeutic strategy for the treatment of patients with glioblastoma multiforme (GBM).

## 2 RESULTS

### 2.1 Clinical correlation between different functional proteins and glioma patients

**FIGURE 1:**
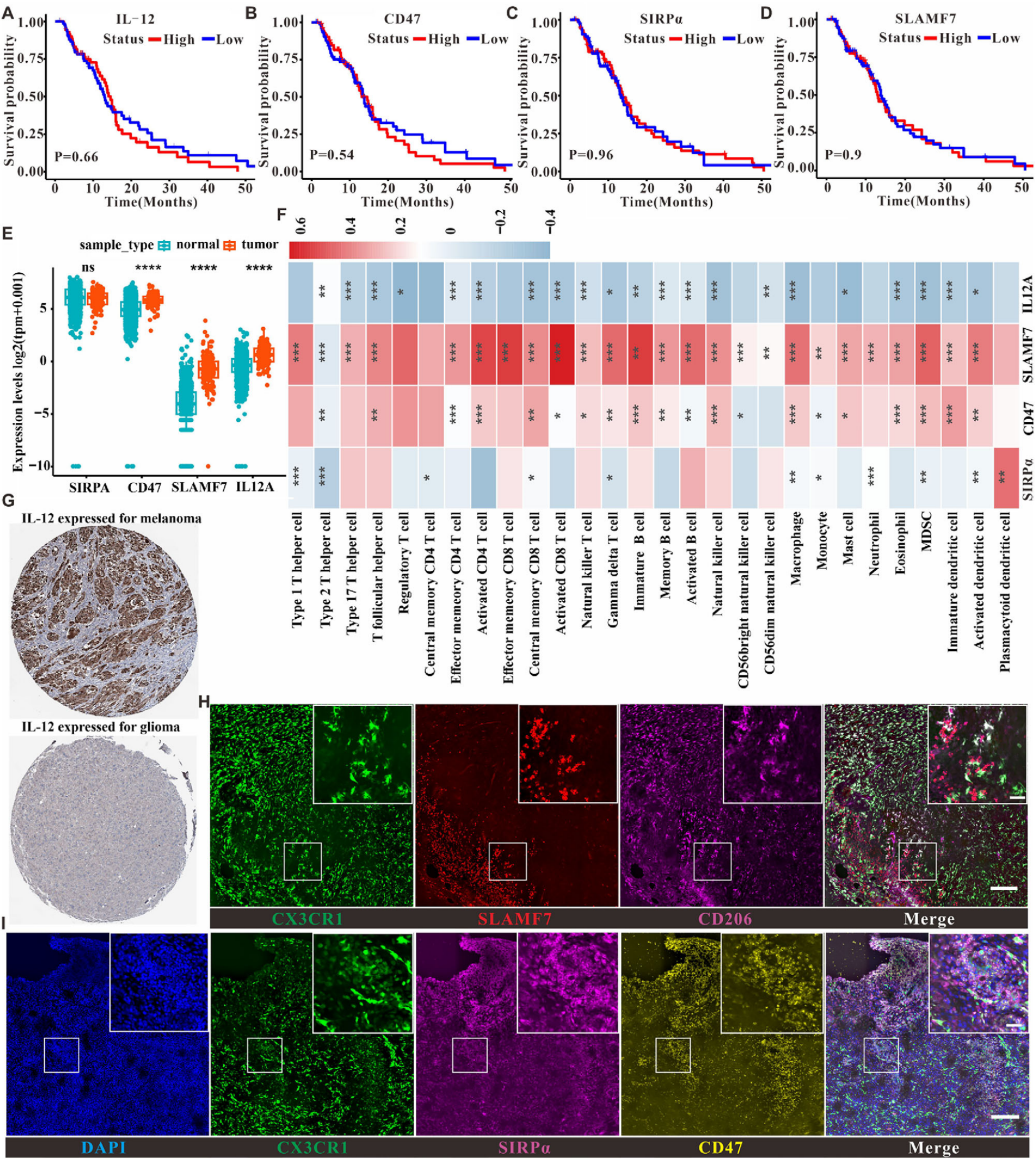
Identification of clinical correlations between different functional proteins and glioma patients. (A) Correlation of IL‐12 with prognostic survival of clinical patients. (B) Correlation of CD47 with prognostic survival of clinical patients. (C) Correlation of SIRPα with prognostic survival of clinical patients. (D) Correlation of SLAMF7 with prognostic survival of clinical patients. (E) Relationship of the expression of different functional proteins in tumors and adjacent cancer. (F) Correlation of different functional proteins with different immune cell infiltration in clinical samples correlation. (G) HPA database‐based analysis of IL‐12 expression in melanoma and glioma patients. (H) Immunofluorescence analysis of SLAMF7 expression in mouse glioma microenvironment, scale bar is 100 µm in the unenlarged figure and 20 µm in the enlarged small figure; (I) Immunofluorescence analysis of CD47 and SIRPα expression in mouse glioma microenvironment, scale bar is the same as that of (H).

By analyzing relevant functional proteins in TCGA_GBM patients with survival software and survminer software, we found that IL‐12, SIRPα, CD47, and SLAMF7 did not significantly affect clinical prognosis (Figure 1A–D). However, the expression levels of IL‐12, CD47, and SLAMF7 were higher in tumors than in adjacent non‐tumorous tissues (Figure 1E), as determined by analyzing their mRNA expression levels in glioma patients using TCGA and GTEx datasets. Furthermore, their expression was strongly correlated with immune cell infiltration (Figure 1F, with the related analysis method described in Section 4). Preclinical studies demonstrated a significant correlation between the regulated expression of these proteins and the survival of a mouse tumor model. Additionally, data from the Human Protein Atlas database indicated that IL‐12 expression in glioma was much lower than in melanoma (Figure 1G), suggesting that the levels of these proteins in glioma patients might be insufficient to modulate changes in the TIME.

To investigate whether the expression of SIRPα, CD47, and SLAMF7 in the mouse glioma model is consistent with that in clinical patients, this study established an in situ glioma model using GL261 cells. Immunofluorescence analysis of tumor sections and non‐tumor sections (Figure S1) showed that SLAMF7 was mainly localized to the tumor periphery, with lower levels in the tumor core (Figure 1H), implying that SLAMF7 expression may hinder glioma development. Moreover, despite the large infiltration of macrophages and microglia secreting abundant SIRPα, CD47 molecules were also highly expressed and colocalized with SIRPα in the TIME (Figure 1I), illustrating a typical immunosuppressive interaction between SIRPα and CD47 molecules. Overall, these findings confirm that the GL261 glioma model has a protein expression profile similar to that of clinical patients.

### 2.2 Controllable expression, structural accuracy and targeting of immune cells for IL‐12 and nCD47‐SLAMF7 expressed in MSC

**FIGURE 2:**
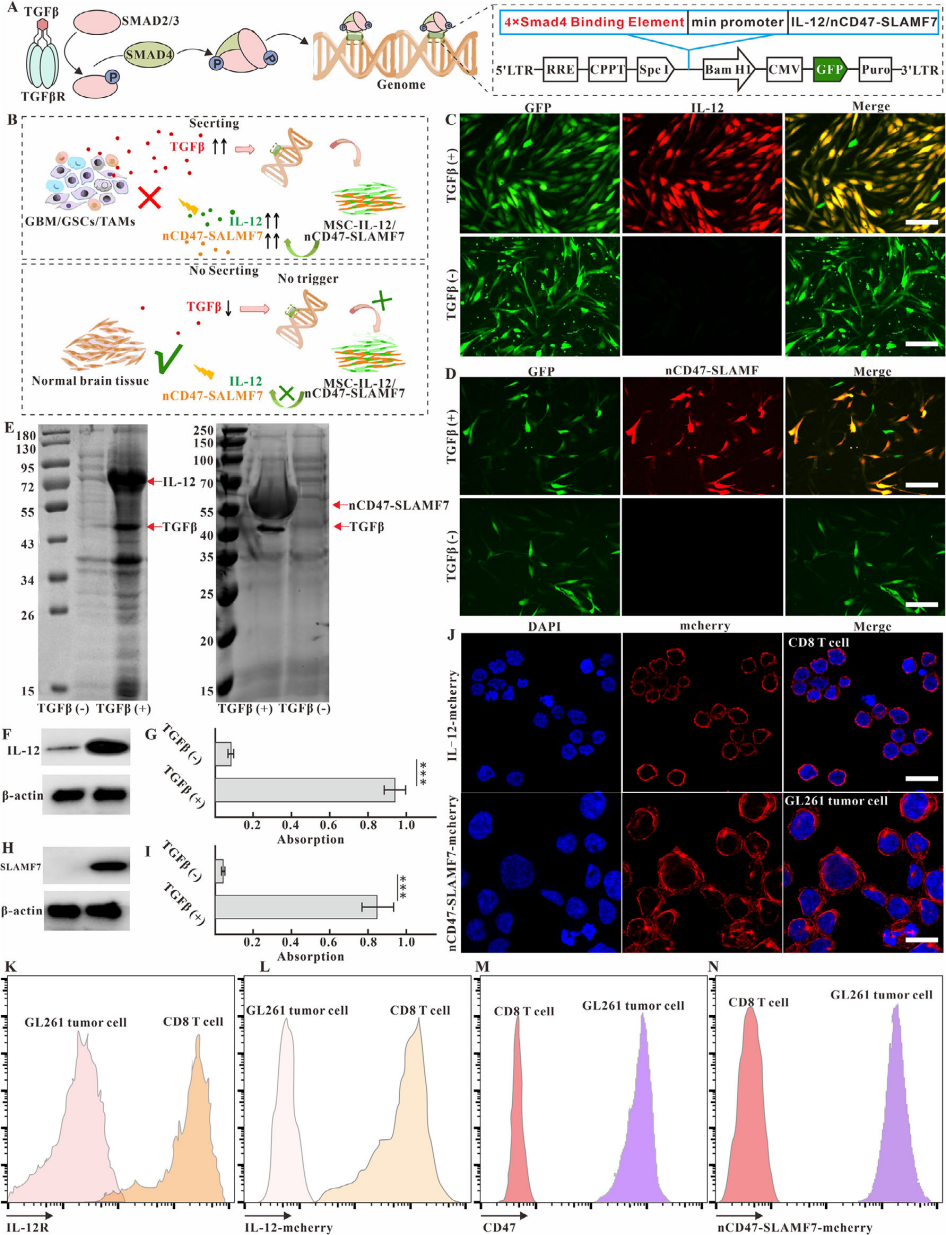
Validation of controlled expression, structural accuracy, and targeting of IL‐12 or nCD47‐SLAMF7 to immune cells in MSC culture medium. (A) Schematic diagram related to the gene regulatory mechanism of MSC releasing IL‐12 or nCD47‐SLAMF7 in response to TGFβ cytokines. (B) Schematic diagram of the mechanism of MSC releasing IL‐12 or nCD47‐SLAMF7 in response to tumor supernatant. (C) Confocal imaging identifies that MSC‐IL‐12 can express IL‐12 cytokines in response to TGFβ. The scale bar is 50 µm. (D) Confocal imaging identifies that MSC‐nCD47‐SLAMF7 expresses nCD47‐SLAMF7 fusion proteins in response to TGFβ. The scale bar is 20 µm. (E) Relative molecular weights of IL‐12 and nCD47‐SLAMF7 secreted by MSC were identified by non‐denaturing SDS‐PAGE gels. (F) Western blotting identification of IL‐12 expression. (G) Elisa's identification of IL‐12 in MSC supernatants. (H) Western blotting identification of nCD47‐SLAMF7 expression. (I) Elisa identification of nCD47‐SLAMF7 in MSC supernatants. (J) Confocal imaging identification of IL −12 or nCD47‐SLAMF7 ability to target T cells or GL261 tumor cells. (K) Flow cytometry identification of IL‐12 receptor expression in CD8 T cells. (L) Flow cytometry identification of IL‐12 cytokine targeting ability to CD8 T cells. (M) Flow cytometry identification of CD47 molecule expression in GL261 cells. (N) Flow cytometry characterization of the targeting ability of nCD47‐SLAMF7 on GL261 cells. Statistical analysis was performed using unpaired t‐tests for (G)and (I). Data are presented as the mean ± SD. *p < 0.05, **p < 0.01, ***p < 0.001.

The side effects of drugs, the presence of the blood‐brain barrier, and the limited concentration of drugs in the tumor microenvironment limit the clinical efficacy of glioma treatment. To overcome these challenges, engineered MSCs with tumor‐homing and TIME‐responsive properties were developed. These cells are driven by transcription factors such as SMAD4 to release IL‐12 or nCD47‐SLAMF7 fusion proteins for glioma cell therapy. SMAD4 is an effector downstream of the TGF‐β receptor and can respond with high sensitivity to TGF‐β and other cytokines in the tumor microenvironment (Figure 2A,B). The nCD47‐SLAMF7 construct includes a signal peptide sequence, a nanobody that targets to shield the function of CD47, a linker containing (GGGSGGGG)2, and the SLAMF7 extracellular structural domain SLAMF7_23‐224_. To characterize the controlled expression of IL‐12 and nCD47‐SLAMF7 in MSCs, the mCherry protein was fused to the C‐terminus of the target proteins. The results in Figure 2C,D showed that adding TGF‐β cytokine to the culture medium could effectively induce the expression of IL‐12 and nCD47‐SLAMF7. The culture medium (CM) of MSC‐IL‐12 or MSC‐nCD47‐SLAMF7 was collected and used for non‐denaturing SDS‐PAGE experiments after incubating with TGF‐β for 24 h. Figure 2E showed that the molecular weights of IL‐12 and nCD47‐SLAMF7 secreted into the MSC‐CM were approximately 70 and 50 kDa, respectively, consistent with the designed proteins. The ELISA results (Figure 2G,I) confirmed that the supernatant secreted by the MSCs contained high levels of IL‐12 or nCD47‐SLAMF7. Immunoblotting experiments confirmed the sequence accuracy of these proteins (Figure 2F,H).

To verify that IL‐12 or nCD47‐SLAMF7 secreted into the CM could effectively target downstream cells, spleen‐derived CD8 T cells (Figure 2K and Figure S2A) with a high expression of the IL‐12 receptor and GL261 cells with high expression of CD47 molecules (Figure 2M and Figure S2C) were used as targets. Confocal imaging results (Figure 2J) showed that IL‐12‐mCherry could effectively bind to the membranes of CD8+ T cells when incubated with the MSC‐IL‐12‐mCherry culture medium, while nCD47‐SLAMF7‐mCherry could also effectively bind to the membranes of GL261 cells when incubated with the supernatant of MSC‐nCD47‐SLAMF7‐mCherry cells. Figure 2L,N (Figure S2B,D) further supported the conclusion regarding the targeting ability of IL‐12 and nCD47‐SLAMF7, as demonstrated by flow cytometry.

### 2.3 Characterizing the functional effects of IL‐12 or nCD47‐SLAMF7 contained in MSC culture medium on CD8 T cells or macrophages

**FIGURE 3:**
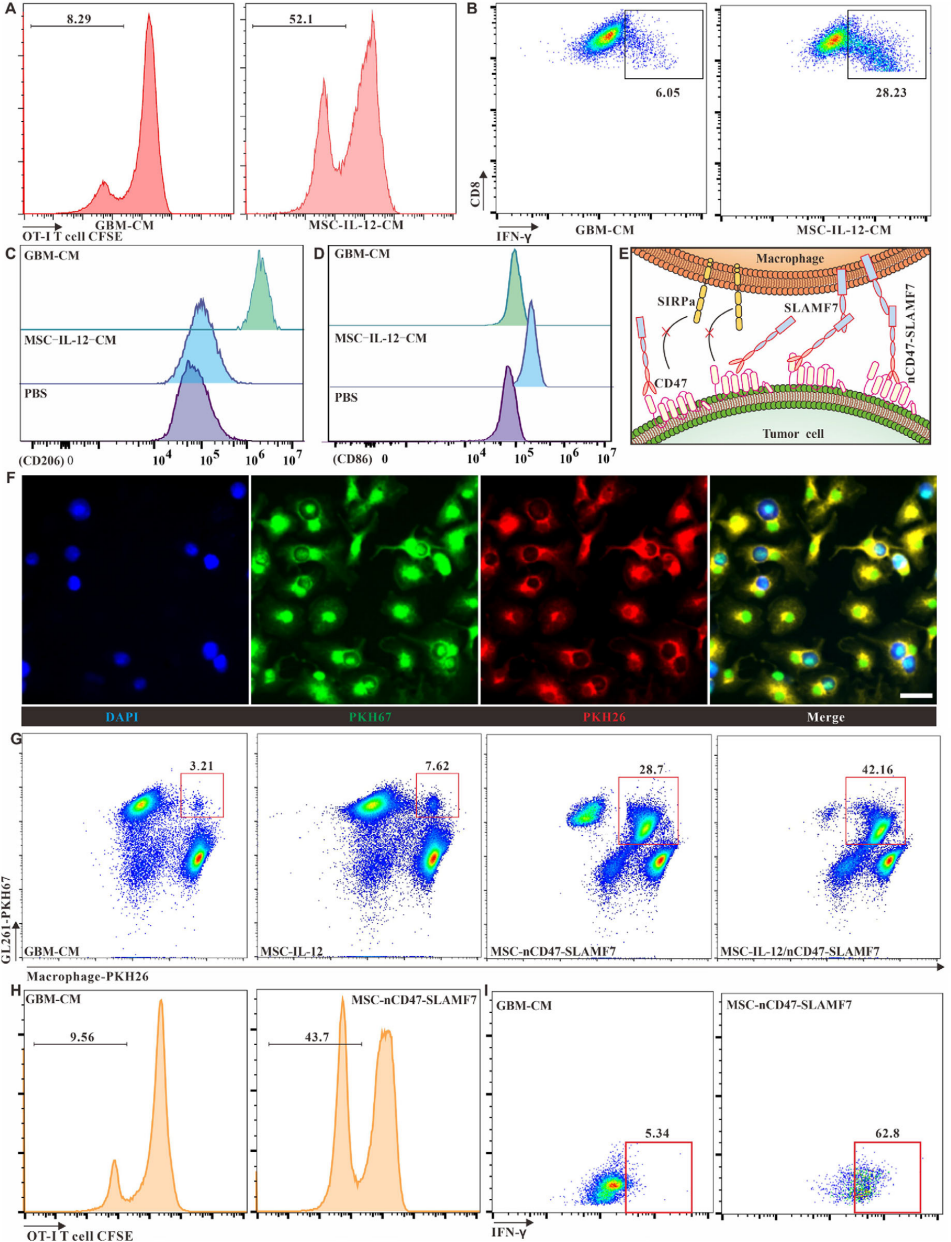
Assessment of the functional impact of IL‐12 or nCD47‐SLAMF7 in MSC supernatants on CD8 T cells and macrophages. (A) Flow cytometric analysis of the proliferation of CD8 T cells in response to GBM‐derived versus MSC‐IL‐12‐derived supernatants. (B) Analysis of CD8 T cell activation and IFN‐γ release when cultured with GBM‐derived or MSC‐IL‐12‐derived supernatants. (C) Determination of CD206 expression on bone marrow‐derived M0 macrophages exposed to GBM‐derived or MSC‐IL‐12 supernatants. (D) Examination of CD86 molecule expression on M0 macrophages in response to different supernatants. (E) Illustration of how nCD47‐SLAMF7 fusion proteins influence macrophage phagocytosis of tumor cells. (F) Confocal microscopy to evaluate the enhancement of glioma cell phagocytosis by macrophages due to MSC‐nCD47‐SLAMF7 supernatants, with a scale bar of 20 µm. (G) Comparative flow cytometry to assess the promotion of tumor cell uptake by macrophages using supernatants from various cellular sources. (H) Investigation of OT‐I T cell proliferation induced by macrophages that have phagocytosed GL261‐OVA^β2m‐/‐^ cells. (I) Analysis of cytokine secretion, such as IFN‐γ, by OT‐I T cells in response to macrophages with internalized GL261‐OVA^β2m‐/‐^ cells.

The main purpose of using MSCs to express IL‐12 and nCD47‐SLAMF7 in this study was to achieve controlled release in the tumor microenvironment as well as effective reversal of the TIME, thus activating both natural and adaptive immunity for effective treatment of glioma. To verify the functional effects of IL‐12 and nCD47‐SLAMF7 on downstream immune cells, we chose OT‐I T cells, which specifically recognize OVA antigens, and bone marrow‐derived macrophages as the subjects of the study. The OT‐I CD8^+^ T cells obtained by magnetic bead sorting were labeled with CFSE dye and then co‐incubated with GL261‐CM or MSC‐CM containing IL‐12 for 48 h followed by the detection of OT‐I T cell proliferation and the expression of IFN‐γ. Figure 3A,B and Figure S3A,B showed that IL‐12‐containing MSC‐CM efficiently induced more T cell proliferation and the production of the effector molecule IFN‐γ compared to GL261‐CM. Furthermore, bone marrow‐derived undifferentiated M0 macrophages were co‐incubated with GL261‐CM or MSC‐IL‐12‐CM for 24 h, and the classical M1‐type marker CD86 and the classical M2‐type marker CD206 was used to label the macrophages. Figure 3C,D and Figure S3C,D showed that GBM‐CM induced high expression of CD206 in M0‐type macrophages and had no effect on the expression of CD86, indicating that they were polarized toward M2‐type macrophages. In contrast, M0‐type macrophages incubated with MSC‐IL12‐CM expressed high levels of CD86 and did not promote the expression of CD206, indicating polarization toward M1‐type macrophages. These results demonstrated that IL‐12 produced by MSC can regulate the development of innate immune cells and adaptive immune cells towards anti‐tumor immunity.

CD47‐SIRPα interactions are well‐established targets in natural immunomodulation, but the systemic toxicity of CD47 antibodies has limited their clinical application. Strategies that allow for the controlled release of CD47 antibodies responsive to the tumor microenvironment have reduced this systemic toxicity. In this study, a CD47 antagonistic nanobody^[^ ^26^ ^]^ was utilized to bind the extracellular region of SLAMF7 molecules, which can modulate the phagocytic activity of phagocyte‐like cells, and the relevant regulatory mechanism is illustrated in Figure 3E. To observe the phenomenon of tumor cell phagocytosis by M0‐type macrophages incubated with MSC‐nCD47‐SLAMF7‐CM, GL261 tumor cells were labeled with PKH67, and M0 macrophages were labeled with PKH26 dyes, respectively. Subsequently, the two cell types were co‐cultured with the addition of MSC‐nCD47‐SLAMF7‐CM. Figure S4A,B and Figure 3F showed that SLAMF alone could enhance the macrophage to engulf tumor cells, and the nCD47‐SLAMF showed the strongest ability to enhance the engulf ability of macrophage. In contrast, neither MSC‐IL12‐CM nor GL261‐CM enhanced the macrophages' phagocytic activity (Figure 3G). Additionally, the culture medium containing both IL‐12 and nCD47‐SLAMF7 had a more pronounced effect on promoting macrophage phagocytosis, which may be due to IL‐12 influencing the polarization state of macrophages.

To establish that macrophages can initiate the adaptive immune system after uptake of tumor cells, OVA‐expressing GL261 cells with knockdown of the β2m gene (GL261‐OVA^β2m‐/−^) were generated by virus transduction. This cell line does not express MHC‐I proteins and cannot present OVA class I antigens on the cell surface, thus preventing the stimulation and proliferation of OT‐I T cells by the tumor cells themselves. We combined the macrophages and GL261‐OVA^β2m‐/−^ tumor cells in a mixed system with GL261‐CM or MSC‐nCD47‐SLAMF7‐CM for 24 h and then co‐incubated with OT‐1 T cells labeled with CFSE. The proliferation of OT‐1 T cells and the release of inflammatory factors such as IFN‐γ after 48 h was examined. Figure 3H,I and Figure S4C,D showed that macrophages that had phagocytosed GL261‐OVA^β2m‐/−^ tumor cells efficiently induced the proliferation of OT‐I T cells and the significant release of the effector molecule IFN‐γ. However, macrophages that phagocytosed tumor cells less efficiently did not produce this effect. These results confirm that nCD47‐SLAMF7 enhances the phagocytic activity of phagocyte‐like cells on tumors and initiates adaptive immune responses.

### 2.4 Identify the tumor‐homing ability of MSC‐IL‐12 or MSC‐nCD47‐SLAMF7

**FIGURE 4:**
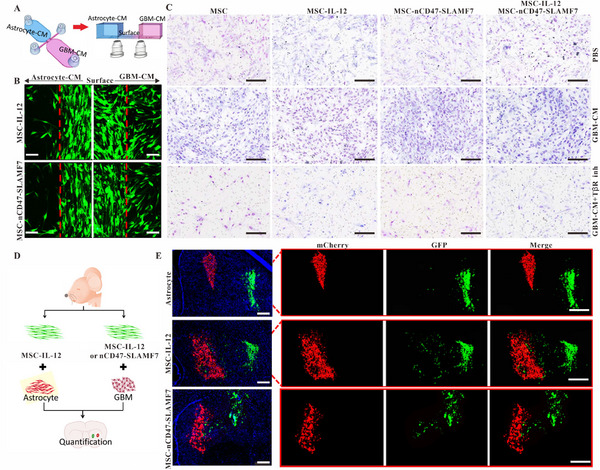
Evaluation of the tumor‐homing capabilities of MSC‐IL‐12 and MSC‐nCD47‐SLAMF7. (A) Illustrative schematic of the personalized Transwell model design. (B) Confocal microscopy shows MSC‐IL‐12 and MSC‐nCD47‐SLAMF7 migrating towards a GBM‐derived supernatant, with a scale bar representing 50 µm. (C) H&E staining demonstrates the homing of MSC‐IL‐12 and MSC‐nCD47‐SLAMF7 in response to a GBM supernatant with or without TGFβ or TGFβ receptor inhibitors; scale bar is 100 µm. (D) Conceptual schematic of the in vivo study designed to validate the tumor‐homing efficiency of MSC‐IL‐12 and MSC‐nCD47‐SLAMF7. (E) Confocal microscopy characterizes the in vivo tumor‐homing of MSC‐IL‐12 and MSC‐nCD47‐SLAMF7, indicated by a scale bar of 100 µm.

The choice of MSC as a carrier for cell therapy strategy in this study lies in the fact that MSC has natural tumor‐homing properties. To ascertain whether overexpression of IL12 or nCD47‐SLAMF7 affects the homing properties of MSCs, a personalized transwell model was designed, and the relevant schematic diagrams are shown in Figure 4A. The surface area was inoculated with MSC expressing the EGFP fluorescent protein, while the left and right compartments were filled with astrocyte‐CM and GL261‐CM, respectively. Figure 4B showed that MSC expressing either IL12 or nCD47‐SLAMF7 have the potential for convergent movement toward the GL261‐CM. To determine whether TGF‐β in the GL261‐CM induced the chemotactic movement of MSC, a TGF‐β receptor inhibitor (LY2109761, 10 ng/mL) was added to the GL261‐CM. H&E staining of the transwell (Figure 4C) demonstrated that GL261‐CM efficiently promoted the infiltration of MSCs, whereas GL261‐CM containing LY2109761 blocked this function, indicating that TGF‐β released by GL261 is the main cytokine‐inducing MSC homing.

To validate the in vivo tumor‐homing effect of MSCs expressing IL‐12 or nCD47‐SLAMF7, we constructed astrocyte and GL261 tumor cell lines expressing mCherry, as well as MSCs expressing IL12‐EGFP and nCD47‐SLAMF7‐EGFP. The schematic diagrams of the injection strategy are shown in Figure 4D. Seven days after in situ injection, the mice's brains were subjected to immunofluorescence staining. Confocal imaging (Figure 4E) revealed that the number of IL‐12‐expressing MSCs homing toward the astrocyte population was minimal, while a relatively large number of cells homed toward the GL261 cell population. MSCs expressing nCD47‐SLAMF7 did not affect the homing ability of the MSCs.

### 2.5 In vivo validation of MSC‐IL‐12 and MSC‐nCD47‐SLAMF7 for glioma treatment

**FIGURE 5:**
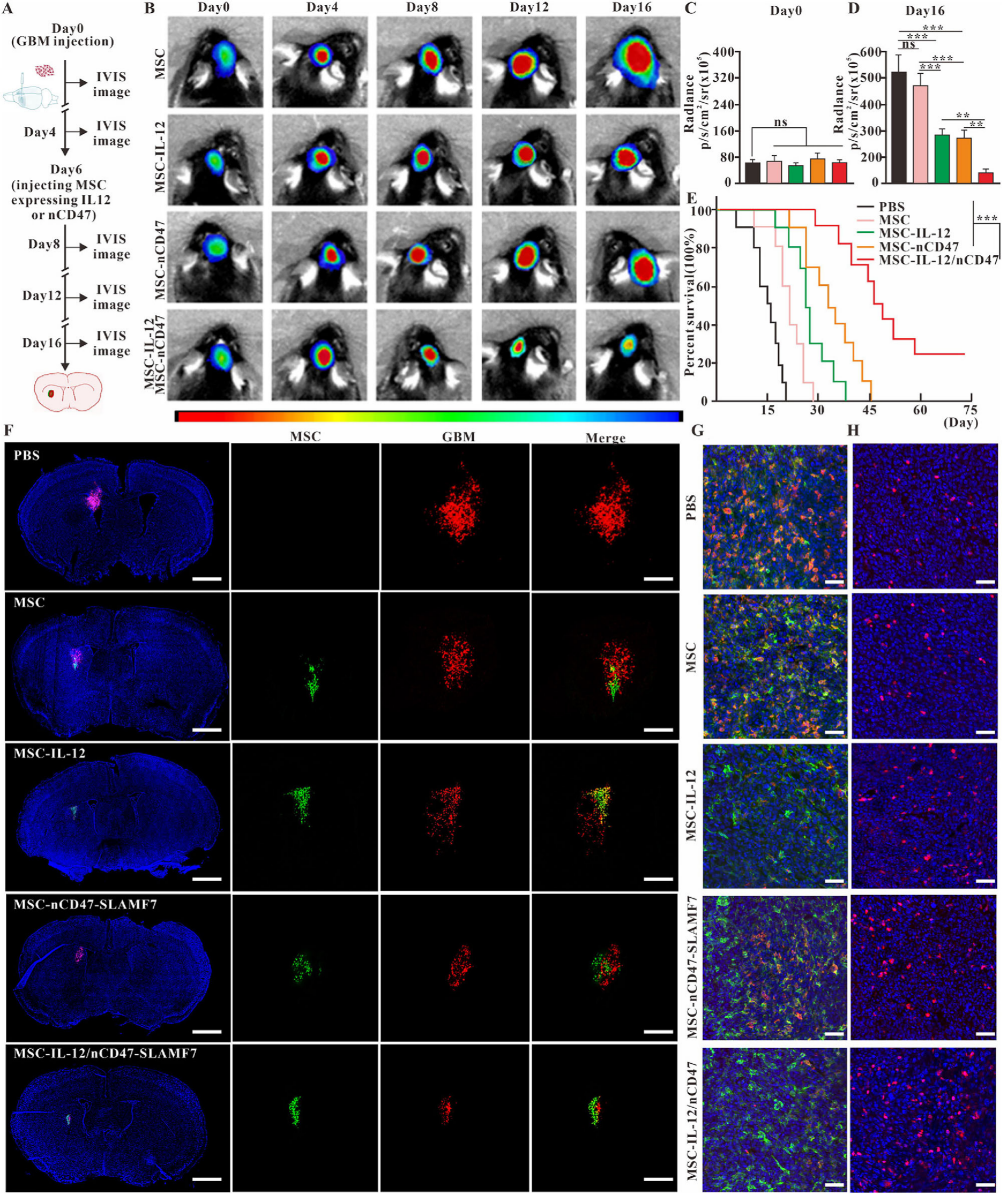
In vivo validation of the therapeutic potential of MSC‐IL‐12 and MSC‐nCD47‐SLAMF7 against glioma. (A) Diagram illustrating the treatment process using MSCs engineered to express various functional proteins. (B) Live imaging of small animals to track glioma progression in different treatment groups over time. (C) Measurement of glioma bioluminescence intensity in various treatment groups on day 0. (D) Measurement of glioma bioluminescence intensity in various treatment groups on day 16. (E) Analysis of the survival rates in the GL261 in situ mouse model (*n* = 10 for each group). (F) Immunofluorescence to visualize the localization of MSCs expressing distinct functional proteins at the glioma site. Scale bar: 1 mm (left panel), 200 µm (right panel). (G) Immunofluorescence to demonstrate macrophage CD206 expression across different treatment groups. Scale bar: 50 µm. (H) Immunofluorescence to assess CD8 T cell infiltration in the various treatment groups. Scale bar: 50 µm. Statistical analysis was performed using one‐way ANOVA with Tukey's multiple comparison test for (C, D), and log‐rank Mantel–Cox test for (E). Data are presented as the mean ± SD. *p < 0.05, **p < 0.01, ***p < 0.001, and ns: not significant.

The previous results have confirmed the successful construction of engineered MSCs and their effective homing to glioma sites. To evaluate if this cell therapy strategy could control the growth of glioma in vivo, we established a mouse glioma model using GL261‐luciferase (GL261‐Luc) cells, following the treatment protocol depicted in Figure 5A. Different mouse groups underwent in vivo small animal imaging, with bioluminescence intensity measured at several time points. The data in Figure 5B indicate that a single injection of MSCs expressing either IL‐12 or nCD47‐SLAMF7, and especially a mixed injection of both cell types on day 6, inhibited glioma growth. Especially, the group receiving the combined cell types displayed the most significant growth inhibition. Bioluminescence intensity analysis on day 0 (Figure 5C) and day 16 (Figure 5D) shows that MSCs expressing both target proteins had a more pronounced inhibitory effect on GBM growth. Survival analysis (Figure 5E), tumor area analysis (Figure S6), and the body weight changes of different groups (Figure S5C) suggest that while MSCs expressing IL‐12 alone or nCD47‐SLAMF7 alone marginally improved survival, the combination treatment notably increased survival rates. However, the combined anti‐tumor treatment effect of the two significantly enhanced the survival rate of the mice. To verify that the engineered MSCs could also be universally applicable for treating other tumor cells that highly express TGF‐β, we selected Lewis tumor cells with high expression of TGF‐β to establish an intracranial tumor model. The results of Figures S5A,B and S5D demonstrated that the simultaneous intracranial injection of MSCs expressing IL12 and MSCs expressing nCD47‐SLAMF7 yielded the most substantial inhibitory effect on the Lewis tumor cells. Additionally, the biosafety of MSCs is a critical factor in their clinical use. According to Figure S5E, different groups of engineered MSCs were either cleared or underwent apoptosis by 8 weeks, with no potential for tumorigenicity. In summary, this study's engineered MSCs present a promising therapeutic approach for tumors characterized by high TGF‐β expression.

To confirm whether the injected MSCs exerted localized effects on the tumor and to monitor tumor growth in vivo, GL261‐mCherry cells were injected on day 0. Different groups of EGFP‐expressing MSCs were then administered on day 6. On day 16, immunofluorescence experiments on the mice allowed observation of the co‐localization of GL261 cells with MSCs. Figure 5F indicated that the population of GL261 cells was the smallest in the group injected with a combination of MSC types, and a high degree of co‐localization between MSCs and GL261 cells was observed across different groups. These observations suggest that MSCs acted primarily within the tumor in situ. The effects on immune cells within the TIME were also observed following injection with the various groups of MSCs.

**FIGURE 6:**
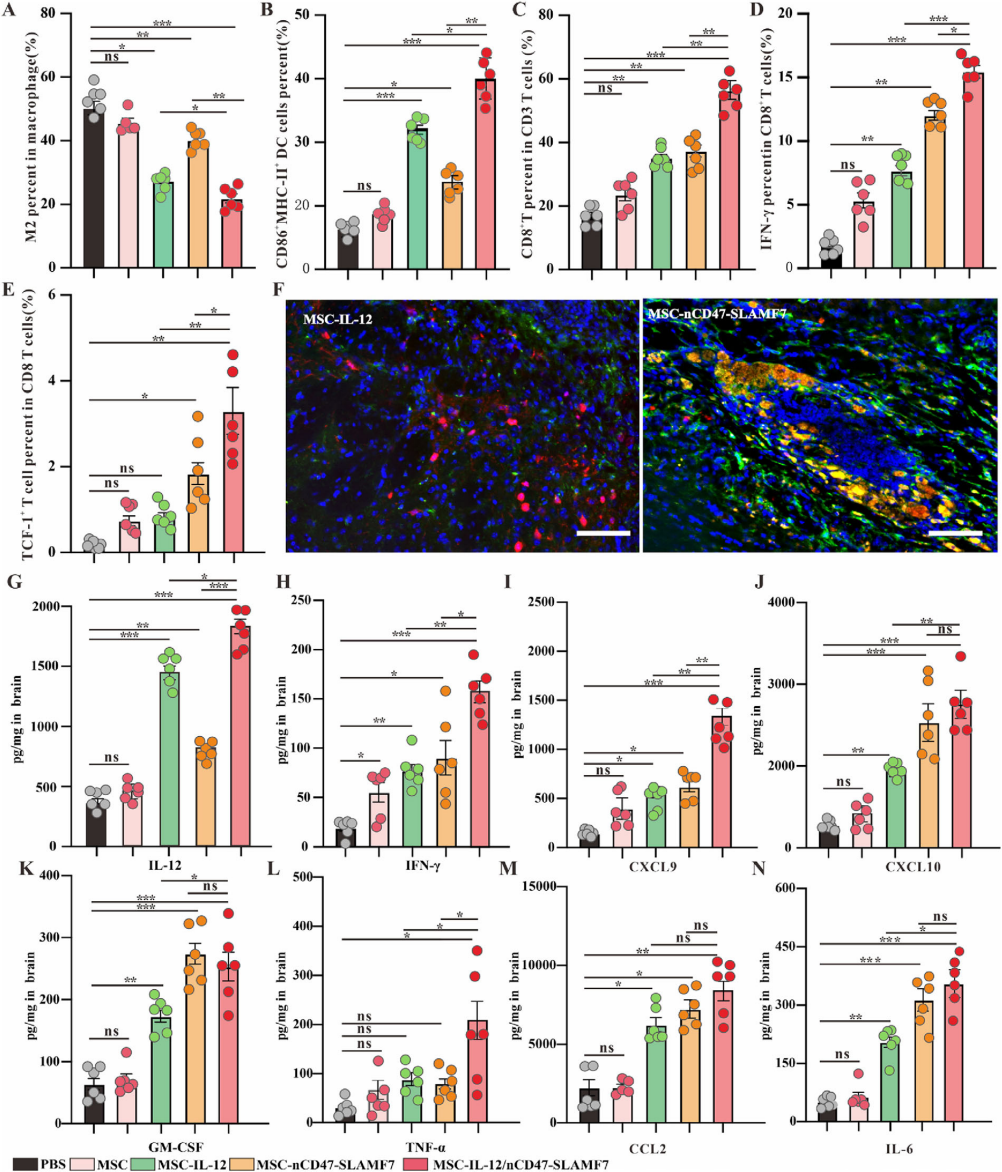
Validation of the ability of MSC‐IL‐12 and MSC‐nCD47‐SLAMF7 to reverse the TIME. (A) Identification of the proportion of M2‐type macrophages in different given treatment groups (*n* = 6 for each group). (B) Identification of the proportion of mature DC cells in different given treatment groups (*n* = 6 for each group). (C) Identification of the proportion of CD8 T cells occupying CD3 T cells in different given treatment groups (*n* = 6 for each group). (D) Identification of the proportion of IFN‐γ‐positive CD8 T cells in different given treatment groups (*n* = 6 for each group). (E) Identification of the proportion of exhausted precursor CD8 T cells in different given treatment groups (*n* = 6 for each group). (F) Immunofluorescence to identify that in vivo injection of MSC‐nCD47‐SLAMF7 promotes the uptake of tumor cells by macrophages, with a scale bar of 100 µm. (G) Identification of IL‐12 content in the lysed supernatant of the mouse brain in different given treatment groups (*n* = 6 for each group). (H) Identification of IFN‐γ content in the lysed supernatant of the mouse brain in different given treatment groups (*n* = 6 for each group). (I) Determination of CXCL9 in mouse brain lysate supernatant in different given treatment groups (*n* = 6 for each group). (J) Determination of CXCL10 in mouse brain lysate supernatant in different given treatment groups (*n* = 6 for each group). (K) Determination of GM‐CSF in mouse brain lysate supernatant in different given treatment groups (*n* = 6 for each group). (L) Determination of TNF‐α in mouse brain lysate supernatant in different given treatment groups (*n* = 6 for each group). (M) Determination of CCL2 in the lysed supernatant of mouse brain in different given treatment groups (*n* = 6 for each group). (N) Determination of IL‐6 in the lysed supernatant of mouse brain in different given treatment groups (*n* = 6 for each group). Statistical analysis was performed using one‐way ANOVA with Tukey's multiple comparison test. Data are presented as the mean ± SD. *p < 0.05, **p < 0.01, ***p < 0.001.

The colocalization results in Figure 5G showed that both normal GL261 tumors and those injected with the normal MSC group contained a large number of M2‐type macrophages (macrophages in green, CD206 signals in red). In contrast, the number of M2‐type macrophages was low in both the MSC‐IL‐12 injection group and the mixed MSC injection group. This indicated that cytokines such as IL‐12 could alter the differentiation status of macrophages. This finding is consistent with the in vitro results of inducing differentiation of M0‐type macrophages toward the M1‐type. However, the injection of MSC‐nCD47‐SLAMF7 did not significantly reduce the differentiation of M2‐type macrophages, the result is also supported by the statistical data in Figure 6A. The observations in Figure 5H, along with the statistical data in Figure 6C, demonstrated that injections of MSC groups expressing IL‐12 or nCD47‐SLAMF7 led to a substantial increase in CD8 T‐cell infiltration compared to the normal MSC group, with the mixed MSC group exhibiting the highest level of CD8 T‐cell infiltration. The gating strategy of immune cells from the GL261 tumor environment is shown in Figure S7A. Collectively, these findings verify that the IL‐12 and nCD47‐SLAMF7 combination can effectively modulate the tumor‐suppressive microenvironment and significantly increase the infiltration of anti‐tumor effector T cells, consequently inhibiting glioma progression.

### 2.6 Validation of the ability of MSC‐IL‐12 and MSC‐nCD47‐SLAMF7 to reverse the TIME

Injections of mixed MSCs expressing various target proteins have effectively inhibited the in‐situ growth of GL261 and significantly influenced macrophage differentiation and CD8 T cell infiltration. The impact on DC cells, functional T cells, and precursor exhausted‐like T cells (a subset exerting a predominantly anti‐tumor effect) was also investigated. Flow cytometry results indicated that the mixed injection of MSCs expressing both IL‐12 and nCD47‐SLAMF7 led to a notable increase in DC cell maturation and activation within the TIME (Figure 6B), induced a greater release of pro‐inflammatory cytokines such as IFN‐γ from effector CD8^+^ T cells (Figure 6D), and enhanced the infiltration of TCF‐1^+^ T cells (Figure 6E), contributing to a more sustained anti‐tumor immune response.

To confirm nCD47‐SLAMF7‐induced phagocytosis of tumor cells by macrophages in vivo, SLAMF7 expression was first assessed by flow cytometry. Results indicated that SLAMF7 was primarily expressed in macrophages of brain tumors (Figure S8), suggesting that the nCD47‐SLAMF7 fusion protein may principally act on macrophages to promote tumor cell uptake. Additionally, immunofluorescence on glioma models injected with MSC‐IL‐12 or MSC‐nCD47‐SLAMF7 was performed. Figure 6F illustrated that in the MSC‐nCD47‐SLAMF7‐injected group, macrophages (marked in green) effectively engulfed GL261 tumor cells (marked in red), unlike macrophages in the MSC‐IL‐12‐injected group.

To further substantiate the impact of mixed MSCs on the TIME, we also examined the expression of cytokines associated with anti‐tumor immunity using a custom multi‐cytokine assay kit. Figures 6G–N demonstrated that, compared to other groups, pro‐inflammatory cytokines such as TNF‐α and IL‐6 were significantly upregulated in the supernatant from the glioma mouse model injected with the mixed MSC group. Additionally, IFN‐γ (associated with CD8 T‐cell activity) and CXCL9 and CXCL10 (associated with T‐cell infiltration) were also markedly elevated, with some increase observed in the MSC‐IL‐12 and MSC‐nCD47‐SLAMF7 groups compared to the control MSC group. Furthermore, cytokines related to macrophage infiltration and differentiation, such as GM‐CSF, CCL2, and IL‐12, were significantly upregulated in the supernatant from both the single MSC injection groups and the mixed injection group, with the latter showing higher levels. These results confirm that a strategic combination of active proteins with MSCs plays a crucial role in modulating the TIME and offers a novel therapeutic approach for the clinical treatment of GBM patients.

## 3 DISCUSSION

In this study, MSCs were used as carriers for a cell therapy strategy, utilizing their tumor‐homing characteristics that respond to TGF‐β to achieve controlled release of bioactive proteins (IL‐12 or nCD47‐SLAMF7) in the tumor microenvironment while avoiding systemic toxicity. By fully activating both innate and adaptive immune cells, the strategy effectively reversed the TIME and delayed the progression of glioma. As the engineered MSCs have shown therapeutic advantages in GL261 and Lewis tumor cells, they may be universal for treatment in all tumor cells that highly express TGF‐β. Overall, this study proposes a novel therapeutic approach for the clinical treatment of GBM patients.

The CD47/SIRPα signaling axis plays a crucial role in promoting the formation of the TIME, wherein CD47 can cis‐bind to the pro‐phagocytosis protein SLAMF7, inhibiting its dependent phagocytosis effects, in addition to trans‐binding to SIRPα^14^. Furthermore, CD47 monoclonal antibody‐mediated phagocytosis requires the SLAMF7 signaling pathway,^[^ ^27^ ^]^ as studies have shown that SIRPα expression in macrophages reduces CCL8 secretion by inhibiting Syk/Btk kinase activity, thereby impeding T cell recruitment and subsequent anti‐tumor responses.^[^ ^28^ ^]^ Besides, CD47 antibody has been used to inhibit the development of glioma and showed no side effects to the nerve system.^[^ ^29^ ^]^ These studies revealed that pairing the CD47 antibody with SLAMF7 might enhance tumor phagocytosis of macrophages rather than combining the SIRPα antibody with SLAMF7. In this study, we opted to use nanobodies instead of traditional antibodies primarily because nanobodies lack the Fc segment, which can effectively reduce the risk of Fc receptor‐mediated inflammatory diseases,^[^ ^30^ ^]^ and their shorter amino acid sequences and simple post‐translational modifications can promote their massive expression in target cells.

The full‐length SLAMF7 protein and CD47 nanobody were initially designed to construct a fusion protein targeting tumor cells. However, the fusion protein could not be transported into the supernatant via the exocytosis signal peptide sequence. The primary reason may be that the α‐helix structure formed by SLAMF7_225‐245_ in the SLAMF7 protein prevents its exocytosis into the MSC supernatant. To enhance the secretion of the nCD47‐SLAMF7 fusion protein, this study utilized SLAMF7_23‐224_ (the extracellular domain of SLAMF7) to bind to the anti‐CD47 nanobody and added signal peptide sequence to its N‐terminal. Figure 2I demonstrates that the use of truncated SLAMF7 can effectively promote the exocytosis of fusion proteins while preserving their ability to modulate tumor phagocytosis by macrophages. This is the first time the use of truncated SLMAF7_23‐224_ has been proposed as a substitute for the function of full‐length SLAMF7 in exerting biological effects.

The disadvantages of cell therapy strategies such as CAR‐T or CAR‐NK include off‐target effects and cytokine storms caused by uncontrolled cell expansion. However, the MSCs used in this study are programmed to undergo apoptosis or differentiate locally into other cell types after 10 generations of propagation, which does not lead to severe cytokine storms (data not shown). Moreover, using MSCs as carriers for cell therapy enables the local delivery of high concentrations of biofunctional proteins and overcomes the blood‐brain barrier through in situ injections, providing a novel therapeutic strategy for the clinical treatment of GBM patients or for preventing the recurrence of GBM after surgery.

## 4 METHODS

### 4.1 Mice

C57BL/6J female mice were obtained from Hunan Slyke Jingda Laboratory Animal Co. Ltd. in Hunan, China. These mice were bred and maintained in a specific pathogen‐free (SPF) barrier facility. All animal studies were conducted under the approval of the Hubei Provincial Animal Care and Use Committee and followed the experimental guidelines established by the Animal Experimentation Ethics Committee of Huazhong University of Science and Technology. This study was approved by the Ethics Committee of Huazhong University of Science and Technology (IRB ID: TJ20170201).

### 4.2 Cell lines

The murine glioma cell line GL261 was purchased from the National Cancer Institute (NCI, Frederick, MD, USA) and cultured in DMEM low glucose (Sigma‐Aldrich, St. Louis, Missouri, USA) supplemented with 10% (v/v) fetal bovine serum (FBS Superior, Sigma‐Aldrich), 1% (v/v) MEM non‐essential amino acid solution (Thermo Fisher Scientific, Waltham, MA, USA), and 1% (v/v) penicillin/streptomycin (Sigma‐Aldrich). Mouse astrocytes were obtained from the China Center for Type Culture Collection (Wuhan, China) and grown in Dulbecco's Modified Eagle's Medium (DMEM) (Gibco, Grand Island, NY, USA) containing 10% Fetal Bovine Serum (FBS) (Gibco, Grand Island, NY, USA) and 1% penicillin/streptomycin solution. Mouse bone marrow‐derived macrophages (BMDMs) were generated as previously described.^[^ ^31^ ^]^ Murine MSCs were purchased from Pro‐cell (Cat number # CP‐M131) and cultured in Complete medium (specifically designed for mouse MSCs, purchased from Pro‐cell) containing 10% (v/v) FBS and 1% (v/v) penicillin/streptomycin. The selection of murine MSC‐IL‐12 or MSC‐nCD47‐SLAMF7 was maintained with 100 pg mL^−1^ G418 (Sigma‐Aldrich). Preparation of MSC for injection into mice was performed as described.^[^ ^30^ ^]^ All cells were maintained in an incubator at 37°C in a humidified atmosphere of 95% and 5% CO_2_. The cell lines were examined for mycoplasma and viruses according to the FELASA guidelines by Charles River Research Animal Diagnostic Services (CR RADS.Wilmington, MA, USA: Mouse essential panel) before in vivo transplantation.

### 4.3 Analysis of the relationship between functional proteins and immune cell infiltration

The bulk RNA‐seq profiles of GBM data and matching normal human tissue data were obtained from UCSC Xena (https://xenabrowser.net/).^[^ ^32^ ^]^ The expression profiles were transferred to transcripts per kilobase million (TPM) format, and the log_2_(TPM+0.001) format data were used for subsequent analysis. Relevant prognostic information was also downloaded from the UCSC Xena database. The prognostic role of relevant genes in TCGA‐GBM was then assessed using the Kaplan–Meier model and univariate Cox regression using the R “packages‐survival”(v3.3.1) (https://CRAN.R‐project.org/package = survival) and “survminer” (v0.4.9). The ssGSEA algorithm in R package “GSVA” (v1.46.0)^[^ ^33^ ^]^ was utilized to quantify the immune cell infiltration of different cancers. The Marker gene set of immune cells came from the reference study.^[^ ^34^ ^]^ The relationship between the expression of IL12A, CD47, SIRPα, SLAMF7 mRNA and 28 immune cell subsets was studied by using the R software package “pheatmap” (v1.0.12), and the heat map was generated by Spearman correlation analysis. All these analyses were performed in R (4.2.2).

### 4.4 Lentiviral production, infection, and identification

GL261‐OVA^β2m‐/−^ was transduced with lentiviral vectors coding for OVA and sh RNA for β2m. Astrocyte‐mCherry cells were transduced with lentiviral vectors coding for mCherry. For both in vitro and in vivo experiments, mouse MSCs were transduced with lentiviral vectors encoding EGFP, EGFP/Fluc, mCherry, or mCherry/Fluc, as well as lentiviral vectors driving the expression of IL‐12 (peptide sequence shown in Table 1) or nCD47‐SLAMF7 (peptide sequence shown in Table 1) in response to TGF‐β^30^. To confirm TGF‐β controlled MSC expressing IL‐12 or nCD47‐SLAMF7, MSC‐IL‐12 or MSC‐nCD47‐SLAMF7 were cultured in MSC complete media in the presence or absence of TGF‐β1 (Abcam) and/or TGF‐β2 (Abcam) at a concentration of 10 ng mL^−1^. IL‐12 or nCD47‐SLAMF7 expression was assessed by Western blot and ELISA (biolegend). The viral vector was packaged from HEK293 cells.

### 4.5 In vitro cellular expression and target ability of IL‐12 or nCD47‐SLAMF7

To determine which MSCs responded to TGF‐β and expressed IL‐12 or nCD47‐SLAMF7, as well as to examine the cellular co‐localization of IL‐12 and nCD47‐SLAMF7 in the culture medium, GL261 cells, MSC‐IL‐12, MSC‐nCD47‐SLAMF7, and CD8 T cells from mouse spleen were seeded in glass‐bottom cell culture dishes (NEST, catalog no. 801001; 1 × 10^5^ cells per well). They were then incubated with a culture medium containing TGF‐β or medium obtained from MSC‐IL‐12 or MSC‐nCD47‐SLAMF7 for 3 h. Subsequently, these cells were washed three times with PBS, fixed in 1% paraformaldehyde for 15 min, and then washed with PBS again. Cellular imaging was performed using confocal laser scanning microscopy (LSM 710). For quantitative assessment of cellular uptake, cells were seeded in six‐well cell culture dishes and treated as described above. They were then washed with PBS three times, collected, fixed, and resuspended in PBS for flow cytometry detection.

### 4.6 Native SDS‐PAGE to verify the IL‐12 and nCD47‐SLAMF7

The procedure was similar to conventional SDS‐PAGE, with the exception that the gel and electrophoresis buffers did not contain SDS. We mixed 5× loading buffer with culture medium obtained from MSC‐IL‐12 or MSC‐nCD47‐SLAMF7 and then added the electrophoretic sample to the gel. Finally, the PAGE gel was stained with Coomassie Brilliant Blue solution.

### 4.7 Western blotting

The cell culture medium was treated with RIPA lysis buffer, and protein separation was carried out on an 8% SDS‐PAGE gel. Subsequently, membrane transfer was performed using a 0.2 µm PVDF membrane (Millipore), followed by antibody incubation. The IL‐12 antibody (Catalog number: 17645‐1‐AP), SLAMF7 antibody (Catalog number: 12905‐1‐AP), and β‐actin antibody (Catalog number: 81115‐1‐RR) were all purchased from Proteintech. All images were acquired using the ChemiDoc Imaging System (Bio‐Rad).

### 4.8 Horizontal chemotactic mobility assay

The experimental design was adapted from the IBIDITM Culture‐Inserts system, which employs two chambers to separate different cell types on opposite sides.^[^ ^35^ ^]^ For in vitro real‐time chemotactic mobility assays, the culture inserts were transferred to a 35‐mm diameter petri dish. The astrocyte culture medium and the GL261 culture medium were seeded in each well of the culture inserts, with a volume of 1 mL per well. After incubation for 1 day, the culture inserts were removed, creating a 500 µm cell‐free gap. The cell culture dish was placed in an incubator providing a constant temperature of 37°C and 5% CO_2_ for 24 h. Subsequently, the cells were fixed with 4% paraformaldehyde and observed using an inverted fluorescence microscope (Zeiss, Oberkochen, Germany).

### 4.9 Animal model experiments and evaluation of therapeutic effects

To establish the GBM model and Lewis tumor cell in the brain, C57BL/6J female mice (6–8 weeks old) were anesthetized with a 1% pentobarbital sodium solution before all surgical procedures. GL261‐luciferase (LUC) cells or Lewis‐luciferase (LUC) cells (1 × 10^6^ cells suspended in 10 µL of PBS) were stereotactically injected into the right ventricle's striatum. The rate of transfection for GL261 was 1 µL/min, and the total volume was 10 µL. Four days after the inoculation with GL261‐luciferase cells, each mouse underwent bioluminescence imaging to ensure the successful and uniform establishment of the GBM model. Subsequently, the mice were randomly divided into five groups: PBS, MSC, MSC‐IL‐12, MSC‐nCD47‐SLAMF7, and MSC‐IL‐12/MSC‐nCD47‐SLAMF7, and were given their respective treatments. The rate of transfection for different MSCs was 1 µL/min, and the total volume was 10 µL. To assess the development of the GBM model, six mice in each group were imaged on the day when all treatments were completed under 1% pentobarbital sodium anesthesia using the Bruker In Vivo MS FX PRO Imager.

### 4.10 Bioluminescence imaging

After anesthetizing C57BL/6J female mice (6–8 weeks old) with 1% pentobarbital sodium, they were intraperitoneally injected with firefly luciferin (150 mg/kg; Sigma‐Aldrich; CAS: 103404‐75‐7). After 15 min, mice were imaged using the Bruker In Vivo MS FX PRO Imager with 3‐min exposure times for acquiring luminescent images.

### 4.11 Tissue multicolor immunofluorescent staining

Tissue multicolor immunofluorescent staining was performed using the OpalTM 7‐Color Manual IHC Kit (NEL811001KT, PerkinElmer). Tumor tissues were fixed, embedded in paraffin, and sectioned with a microtome. The sections were then dewaxed and hydrated routinely. Antigen retrieval was achieved by applying a Tris‐EDTA Buffer solution, and endogenous peroxidases were quenched using 3% H_2_O_2_. Samples were subsequently blocked with normal goat serum. The slides were incubated overnight with the following antibodies: Alexa Fluor 647 anti‐mouse CD206 (MMR) Antibody (Biolegend, cat# 141711), Alexa Fluor 594 anti‐mouse F4/80 Antibody (Biolegend, cat# 123140), PE anti‐mouse CD319 Antibody (Biolegend, cat# 152005), Alexa Fluor 594 anti‐mouse CD172a Antibody (Biolegend, cat# 144020), and Alexa Fluor 647 anti‐mouse CD47 Antibody (Biolegend, cat# 127509). Subsequently, DAPI was applied for 20 min at room temperature. Finally, tissue immunofluorescence was analyzed using the PE Vectra (PerkinElmer).

### 4.12 Collection of tumor‐infiltrating immunocytes

Tumor‐infiltrating immune cells were obtained from the GBM model as previously described.^[^ ^36^ ^]^

### 4.13 Flow cytometry

For cell‐surface analysis, cells were stained with the anti‐mouse Zombie NIR Fixable Viability Kit (423106) and incubated with antibodies against CD45 (103114), CD11b (101205), F4/80 (123121), CD3 (100212), CD4 (100408), and CD8a (100752) at the recommended concentrations. Incubation was carried out at 4°C for 30 min. For T‐cell intracellular IFN‐γ (505808) cytokine staining, cells were fixed and permeabilized after stimulation with Phorbol 12‐myristate 13‐acetate (PMA) (ab120297, Abcam, 100 ng/mL), Monensin sodium salt (ab120499, Abcam, 1 ug/mL), and Ionomycin calcium salt (5608212, PeproTech, 100 ng/mL) for 3 h. For CD206 (141706) and TCF‐1 (655203) staining, cells were also fixed and permeabilized. All flow cytometry antibodies were purchased from Biolegend (San Diego, CA, USA).

### 4.14 Cytokine detection

The supernatant from mouse GBM model tissue fragmentation was collected for cytokine detection. The LEGENDplex Mouse Cytokine Release Syndrome Panel (13‐plex) with VBottom Plate (purchased from Biolegend).

### 4.15 Statistical analysis

The unpaired two‐tailed Student's *t*‐test to compare the differences between the two groups was used, while survival rates were evaluated with the log‐rank Mantel–Cox test using GraphPad Prism 7 software. Repeated measurements of tumor volume growth were compared using a one‐way analysis of variance (ANOVA). Flow cytometry data were analyzed using FlowJo.10. Significant differences between the groups are indicated by *p < 0.05, **p < 0.01, and ***p < 0.001, and NS, not significant.

## CONFLICT OF INTEREST STATEMENT

The authors declare no conflicts of interest.

## Supporting information

SUPPORTING INFORMATION

## Data Availability

The authors declare that all data supporting the results of this study are available in the paper and supporting information. Source data are provided in this paper.
