# Supplementary material for: Bioengineer mesenchymal stem cell for treatment of glioma by IL‐12 mediated microenvironment reprogramming and nCD47‐SLAMF7 mediated phagocytosis regulation of macrophages
Source: Exploration (Beijing). 2024 Jun 25;4(6):20240027. doi: 10.1002/EXP.20240027 (PMC11657999; doi:10.1002/EXP.20240027)
Supplement: Supplementary file 1 — SUPPORTING INFORMATION [file EXP2-4-20240027-s001.docx]

**Supporting information**

**Bioengineer mesenchymal stem cell for Treatment of glioma by IL-12 mediated microenvironment reprogramming and nCD47-SLAMF7 mediated phagocytosis regulation of macrophages**

Man Li^1,2*^, Lisen Lu^3*^, Qungen Xiao^2^, Ali Abdi Maalim^2^, Bin Nie^1^, Yanchao Liu^2^, Ulf D Kahlert ^4^, Kai Shu^2^, Ting Lei^2^, Mingxin Zhu^2^

1. Department of Anesthesiology and Pain Medicine, Hubei Key Laboratory of Geriatric Anesthesia and Perioperative Brain Health, and Wuhan Clinical Research Center for Geriatric Anesthesia, Tongji Hospital, Tongji Medical College, Huazhong University of Science and Technology.

2. Department of Neurosurgery, Tongji Hospital, Tongji Medical College, Huazhong University of Science and Technology, Wuhan, People’s Republic of China

3. College of Biomedicine and Health and College of Life Science and Technology, Huazhong Agricultural University, Wuhan, 430070, China.

4. Molecular and Experimental Surgery, Clinic for General-, Visceral-, Vascular and Transplant Surgery, Faculty of Medicine and University Hospital Magdeburg, Otto-von-Guericke University, Magdeburg, Germany.

* Man Li and Lisen Lu contributed equally to this work.

Corresponding Author:

Mingxin Zhu, Department of Neurosurgery, Tongji Hospital, Tongji Medical College, Huazhong University of Science and Technology, Wuhan, Hubei, People’s Republic of China. Email: [mxzhu@tjh.tjmu.edu.cn](mailto:mxzhu@tjh.tjmu.edu.cn)

Table1. the protein sequence used in the study.

| Protein name | Peptide sequence |
| --- | --- |
| IL-12  (P35-P2A-P40) | MCQSRYLLFLATLALLNHLSLARVIPVSGPARCLSQSRNLLKTTDDMVKTAREKLKHYSCTAEDIDHEDITRDQTSTLKTCLPLELHKNESCLATRETSSTTRGSCLPPQKTSLMMTLCLGSIYEDLKMYQTEFQAINAALQNHNHQQIILDKGMLVAIDELMQSLNHNGETLRQKPPVGEADPYRVKMKLCILLHAFSTRVVTINRVMGYLSSAGSGATNFSLLKQAGDVEENPGPGSGMCPQKLTISWFAIVLLVSPLMAMWELEKDVYVVEVDWTPDAPGETVNLTCDTPEEDDITWTSDQRHGVIGSGKTLTITVKEFLDAGQYTCHKGGETLSHSHLLLHKKENGIWSTEILKNFKNKTFLKCEAPNYSGRFTCSWLVQRNMDLKFNIKSSSSSPDSRAVTCGMASLSAEKVTLDQRDYEKYSVSCQEDVTCPTAEETLPIELALEARQQNKYENYSTSFFIRDIIKPDPPKNLQMKPLKNSQVEVSWEYPDSWSTPHSYFSLKFFVRIQRKKEKMKETEEGCNQKGAFLVEKTSTEVQCKGGNVCVQAQDRYYNSSCSKWACVPCRVRS |
| Mcherry | MVSKGEEDNMAIIKEFMRFKVHMEGSVNGHEFEIEGEGEGRPYEGTQTAKLKVTKGGPLPFAWDILSPQFMYGSKAYVKHPADIPDYLKLSFPEGFKWERVMNFEDGGVVTVTQDSSLQDGEFIYKVKLRGTNFPSDGPVMQKKTMGWEASSERMYPEDGALKGEIKQRLKLKDGGHYDAEVKTTYKAKKPVQLPGAYNVNIKLDITSHNEDYTIVEQYERAEGRHSTGGMDELYK |
| IL-12- mcherry | MCQSRYLLFLATLALLNHLSLARVIPVSGPARCLSQSRNLLKTTDDMVKTAREKLKHYSCTAEDIDHEDITRDQTSTLKTCLPLELHKNESCLATRETSSTTRGSCLPPQKTSLMMTLCLGSIYEDLKMYQTEFQAINAALQNHNHQQIILDKGMLVAIDELMQSLNHNGETLRQKPPVGEADPYRVKMKLCILLHAFSTRVVTINRVMGYLSSAGSGATNFSLLKQAGDVEENPGPGSGMCPQKLTISWFAIVLLVSPLMAMWELEKDVYVVEVDWTPDAPGETVNLTCDTPEEDDITWTSDQRHGVIGSGKTLTITVKEFLDAGQYTCHKGGETLSHSHLLLHKKENGIWSTEILKNFKNKTFLKCEAPNYSGRFTCSWLVQRNMDLKFNIKSSSSSPDSRAVTCGMASLSAEKVTLDQRDYEKYSVSCQEDVTCPTAEETLPIELALEARQQNKYENYSTSFFIRDIIKPDPPKNLQMKPLKNSQVEVSWEYPDSWSTPHSYFSLKFFVRIQRKKEKMKETEEGCNQKGAFLVEKTSTEVQCKGGNVCVQAQDRYYNSSCSKWACVPCRVRSGGGGSGGGGSGGGGSGGGGSMVSKGEEDNMAIIKEFMRFKVHMEGSVNGHEFEIEGEGEGRPYEGTQTAKLKVTKGGPLPFAWDILSPQFMYGSKAYVKHPADIPDYLKLSFPEGFKWERVMNFEDGGVVTVTQDSSLQDGEFIYKVKLRGTNFPSDGPVMQKKTMGWEASSERMYPEDGALKGEIKQRLKLKDGGHYDAEVKTTYKAKKPVQLPGAYNVNIKLDITSHNEDYTIVEQYERAEGRHSTGGMDELYK |
| CD47 nanobody  (VL-Linker-VH) | DIVMTQSPATLSVTPGDRVSLSCRASQTISDYLHWYQQKSHESPRLLIKFASQSISGIPSRFSGSGSGSDFTLSINSVEPEDVGVYYCQNGHGFPRTFGGGTKLEIKGGGGSGGGGSGGGGSGGGGSEVQLVESGGDLVKPGGSLKLSCAASGFTFSGYGMSWVRQTPDKRLEWVATITSGGTYTYYPDSVKGRFTISRDNAKNTLYLQIDSLKSEDTAIYFCARSLAGNAMDYWGQGTSVTVSS |
| SLAMF7_23-224_ | SGTLKKVAGALDGSVTFTLNITEIKVDYVVWTFNTFFLAMVKKDGVTSQSSNKERIVFPDGLYSMKLSQLKKNDSGAYRAEIYSTSSQAS LIQEYVLHVY KHLSRPKVTI DRQSNKNGTCVINLTCSTDQ DGENVTYSWKAVGQGDNQFHDGATLSIAWRSGEKDQALTCMARNPVSNSFSTPVFPQKLC EDAATDLTSLRG |
| nCD47-SLAMF7 | MASPLTRFLSLNLLLLGESIILGSGEADIVMTQSPATLSVTPGDRVSLSCRASQTISDYLHWYQQKSHESPRLLIKFASQSISGIPSRFSGSGSGSDFTLSINSVEPEDVGVYYCQNGHGFPRTFGGGTKLEIKGGGGSGGGGSGGGGSGGGGSEVQLVESGGDLVKPGGSLKLSCAASGFTFSGYGMSWVRQTPDKRLEWVATITSGGTYTYYPDSVKGRFTISRDNAKNTLYLQIDSLKSEDTAIYFCARSLAGNAMDYWGQGTSVTVSSGGGSGGGGGGGSGGGGSGTLKKVAGALDGSVTFTLNITEIKVDYVVWTFNTFFLAMVKKDGVTSQSSNKERIVFPDGLYSMKLSQLKKNDSGAYRAEIYSTSSQASLIQEYVLHVY KHLSRPKVTIDRQSNKNGTCVINLTCSTDQDGENVTYSWKAVGQGDNQFHDGATLSIAWRSGEKDQALTCMARNPVSNSFSTPVFPQKLC EDAATDLTSLRG |
| nCD47-SLAMF7-mcherry | MASPLTRFLSLNLLLLGESIILGSGEADIVMTQSPATLSVTPGDRVSLSCRASQTISDYLHWYQQKSHESPRLLIKFASQSISGIPSRFSGSGSGSDFTLSINSVEPEDVGVYYCQNGHGFPRTFGGGTKLEIKGGGGSGGGGSGGGGSGGGGSEVQLVESGGDLVKPGGSLKLSCAASGFTFSGYGMSWVRQTPDKRLEWVATITSGGTYTYYPDSVKGRFTISRDNAKNTLYLQIDSLKSEDTAIYFCARSLAGNAMDYWGQGTSVTVSSGGGSGGGGGGGSGGGGSGTLKKVAGALDGSVTFTLNITEIKVDYVVWTFNTFFLAMVKKDGVTSQSSNKERIVFPDGLYSMKLSQLKKNDSGAYRAEIYSTSSQASLIQEYVLHVY KHLSRPKVTIDRQSNKNGTCVINLTCSTDQDGENVTYSWKAVGQGDNQFHDGATLSIAWRSGEKDQALTCMARNPVSNSFSTPVFPQKLCEDAATDLTSLRGGGGGSGGGGSGGGGSGGGGSMVSKGEEDNMAIIKEFMRFKVHMEGSVNGHEFEIEGEGEGRPYEGTQTAKLKVTKGGPLPFAWDILSPQFMYGSKAYVKHPADIPDYLKLSFPEGFKWERVMNFEDGGVVTVTQDSSLQDGEFIYKVKLRGTNFPSDGPVMQKKTMGWEASSERMYPEDGALKGEIKQRLKLKDGGHYDAEVKTTYKAKKPVQLPGAYNVNIKLDITSHNEDYTIVEQYERAEGRHSTGGMDELYK |


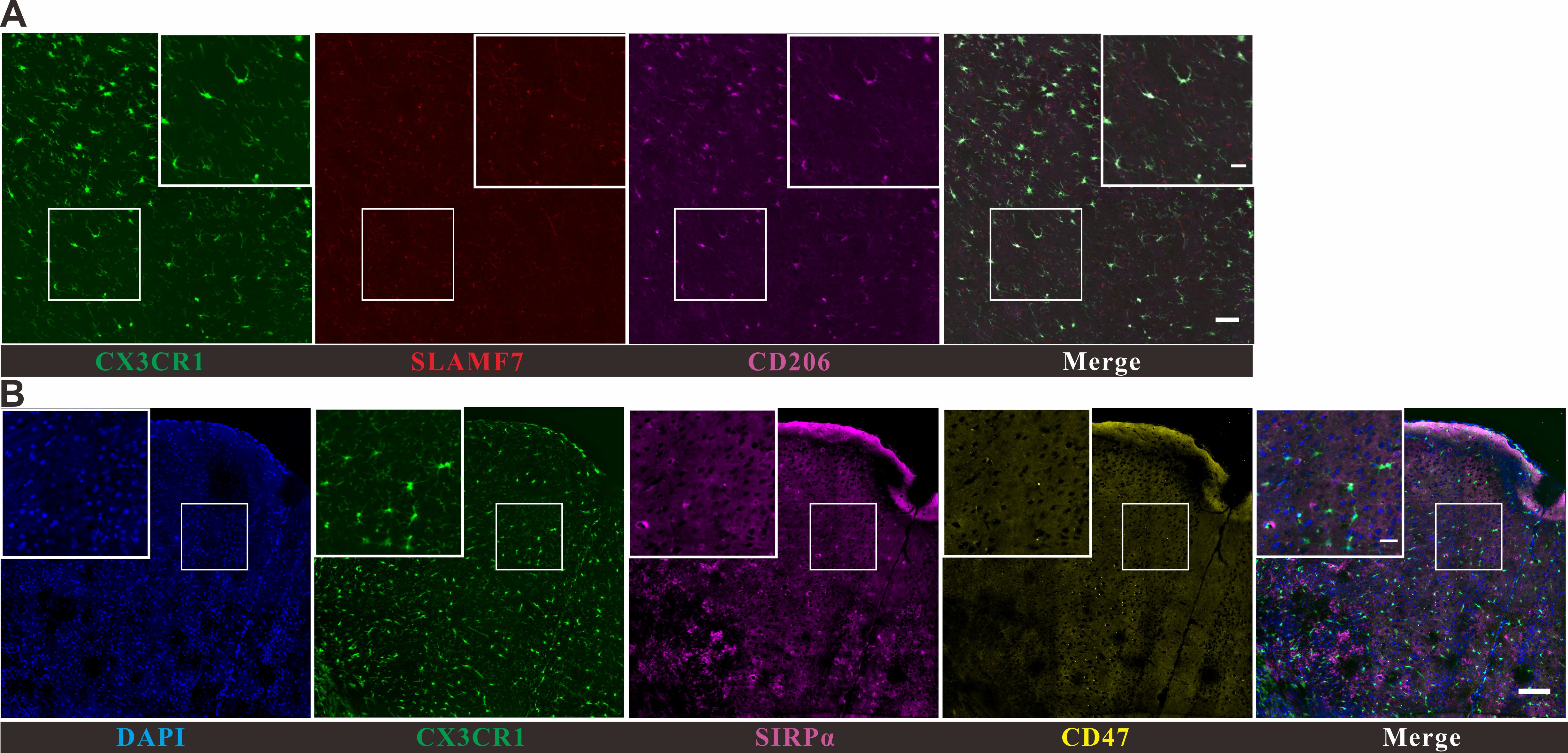


Figure S1. Immunofluorescence analysis of CD47, SLAMF7, CD206 and SIRPα expression in mouse non-tumor microenvironment. (A) Immunofluorescence analysis of SLAMF7 and CD206 expression in C57BL/6J mouse non-tumor microenvironment, scale bar is 100 μm in the unenlarged figure and 20 μm in the enlarged small figure; (B) Immunofluorescence analysis of CD47 and SIRPα expression in mouse non-tumor microenvironment, scale bar is the same as that of (A).


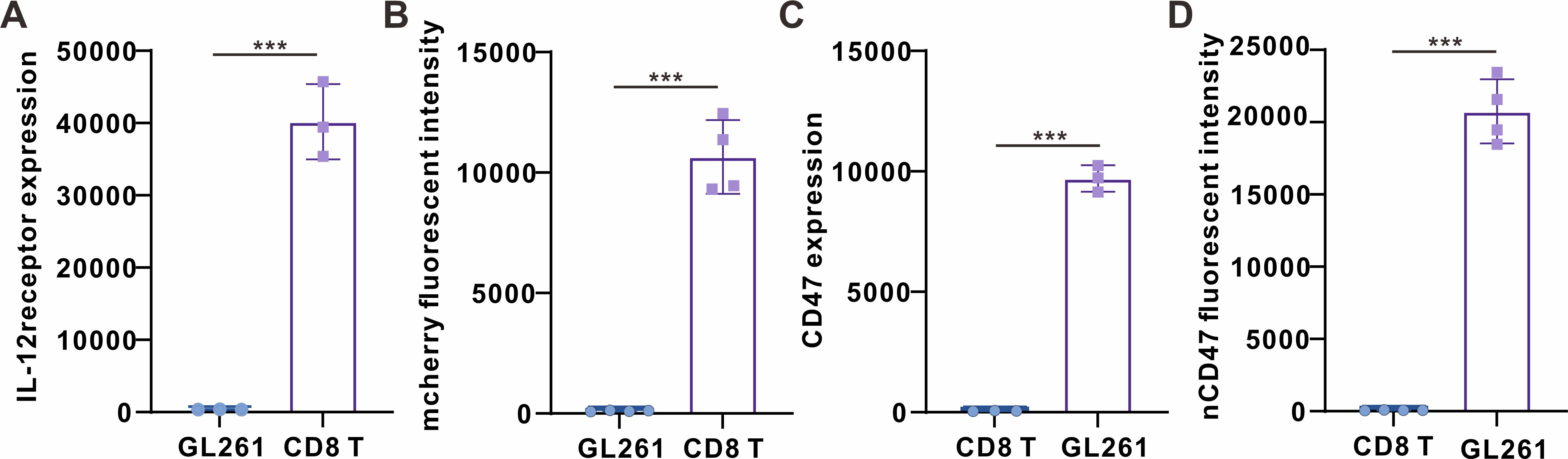


Figure S2. Statistics of the flow cytometry data for IL-12 receptor expression and CD47 expression in GL261 tumor cell and CD8 T cells, and the targeting ability of the IL-12 and nCD47-SLAMF7 secreted by MSCs.(A) Statistics graph of IL-12 receptor expression in CD8 T cells. (B) Statistics graph of IL-12 cytokine targeting ability to CD8 T cells. (C) Statistics graph of CD47 molecule expression in GL261 cells. (D) Statistics graph of the targeting ability of nCD47-SLAMF7 on GL261 cells. . Statistical analysis was performed using unpaired t-test. The data are presented as the mean ± SD (n = 3). *P < 0.05, **P < 0.01, ***P < 0.001.


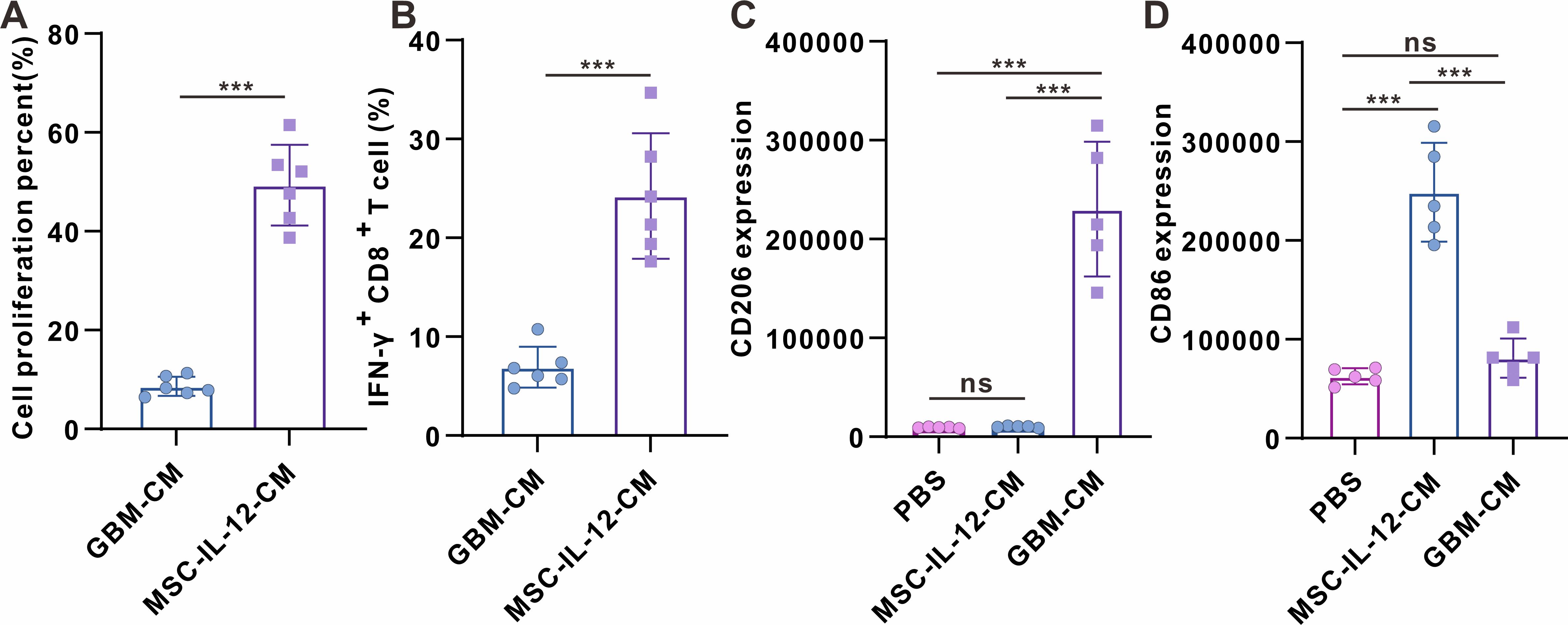


Figure S3. Statistics of the functional impact of IL-12 in MSC supernatants on CD8 T cells and macrophages.(A) Statistics graph of the proliferation of CD8 T cells in response to GBM-derived versus MSC-IL-12-derived supernatants. (B) Statistics graph of CD8 T cell activation and IFN-γ release when cultured with GBM-derived or MSC-IL-12-derived supernatants. (C) Statistics graph of CD206 expression on bone marrow-derived M0 macrophages exposed to GBM-derived or MSC-IL-12 supernatants. (D) Statistics graph of CD86 molecule expression on M0 macrophages in response to different supernatants. Statistical analysis was performed using unpaired t-test for (A )and (B), one-way ANOVA with Tukey's multiple comparison test was performed for (C) and (D). Data are presented as the mean ± SD. *P < 0.05, **P < 0.01,***P < 0.001.


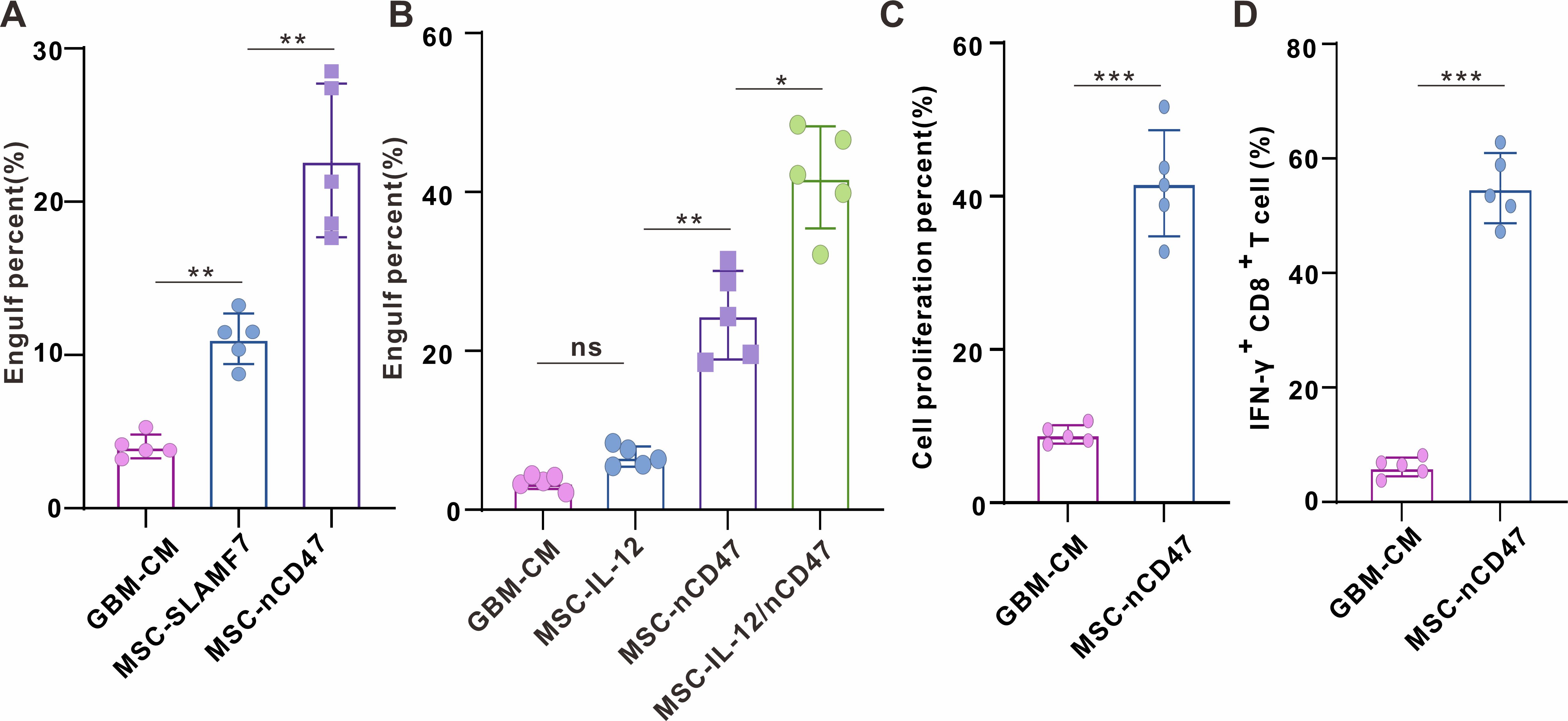


Figure S4.Statistics of the functional impact of nCD47-SLAMF7 in MSC supernatants on CD8 T cells and macrophages. (A) Statistics graph of eugulf efficacy to assess the promotion of tumor cell uptake by macrophages using supernatants from MSC-SLAMF7 or MSC-nCD47. (B) Statistics graph of eugulf efficacy to assess the promotion of tumor cell uptake by macrophages using supernatants from various cellular sources. (C) Statistics graph of OT-I T cell proliferation induced by macrophages that have phagocytosed GL261-OVA^β2m-/-^ cells. (D) Statistics graph of cytokine secretion, such as IFN-γ, by OT-I T cells in response to macrophages with internalized GL261-OVA^β2m-/-^ cells. Statistical analysis was performed using unpaired t-test for (C)and (D), one-way ANOVA with Tukey's multiple comparison test was performed for (A) and (B). Data are presented as the mean ± SD. *P < 0.05, **P < 0.01,***P < 0.001.


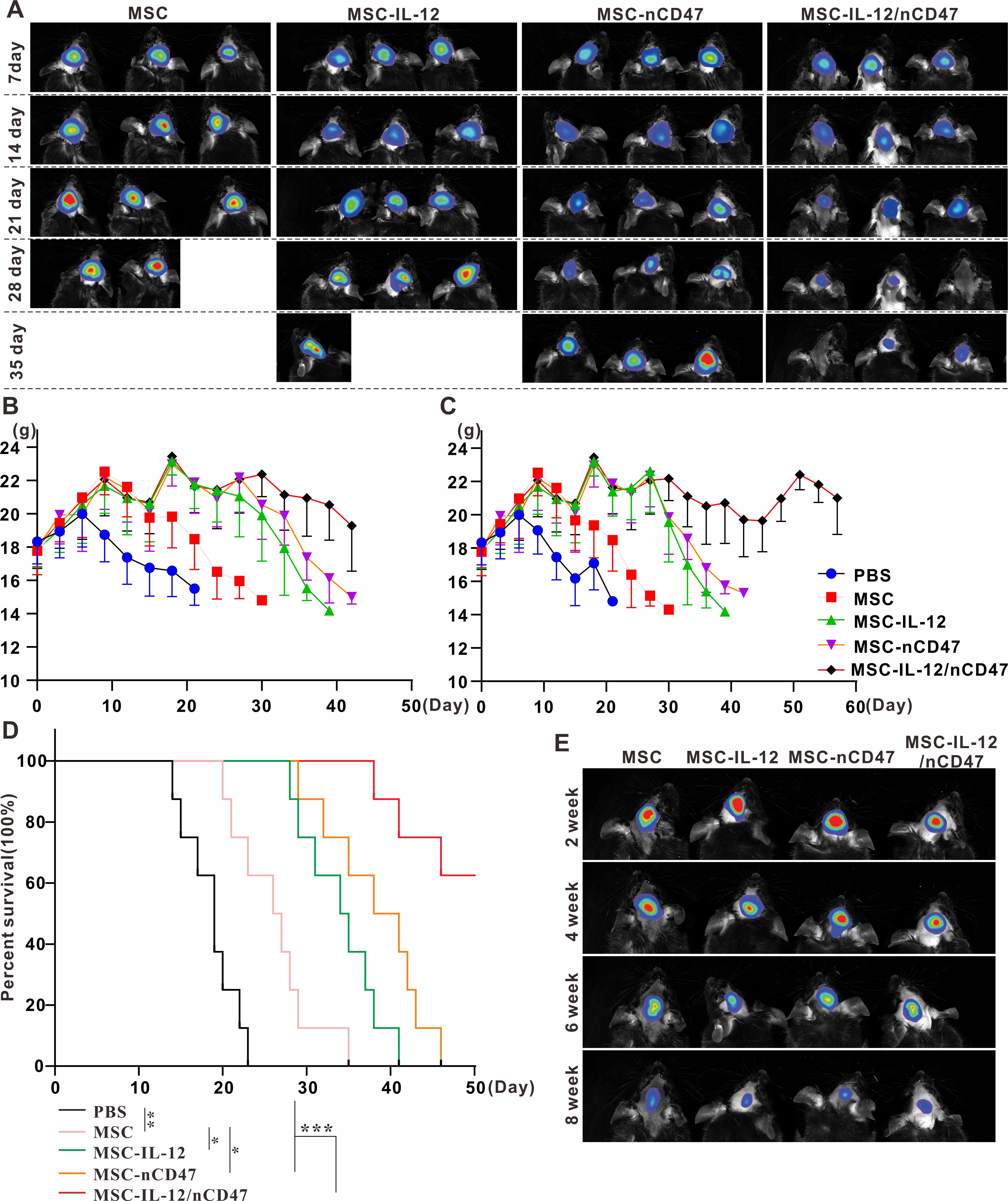


Figure S5.In vivo validation of the therapeutic potential of MSC-IL-12 and MSC-nCD47-SLAMF7 against lewis tumor cell in brain. (A) Live imaging of small animals to track Lewis tumor cell progression in different treatment groups over time. (B) Statistics graph of body weight changes with intracranial injection of lewis tumor cell. (C) Statistics graph of body weight changes with intracranial injection of GL261 tumor cell (n=10). (D) Analysis of the survival rates after intracranial injection of lewis tumor cell (n=10). (E) Live imaging of small animals to track the MSC survival time in different groups over time. Statistical analysis was performed using log-rank Mantel-Cox test. Data are presented as the mean ± SD. *P < 0.05,**P < 0.01, ***P < 0.001, and ns: not significant.


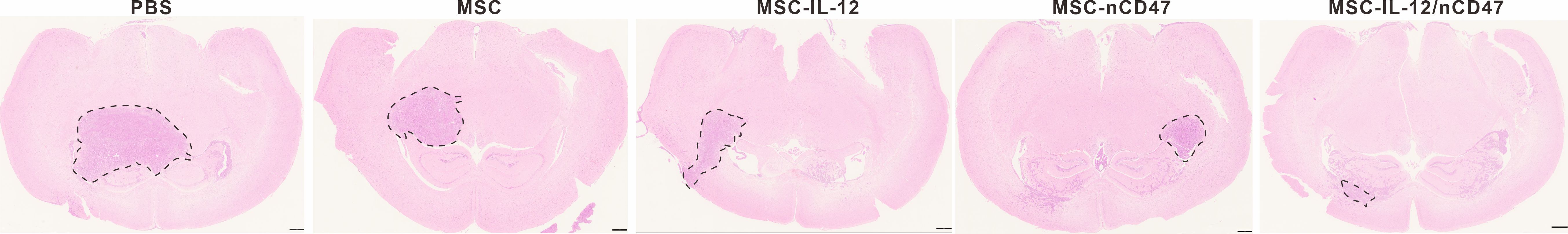


Figure S6. HE staining of brain slice of GL261 tumor model given different treatments. Scale bar: 100 μm.


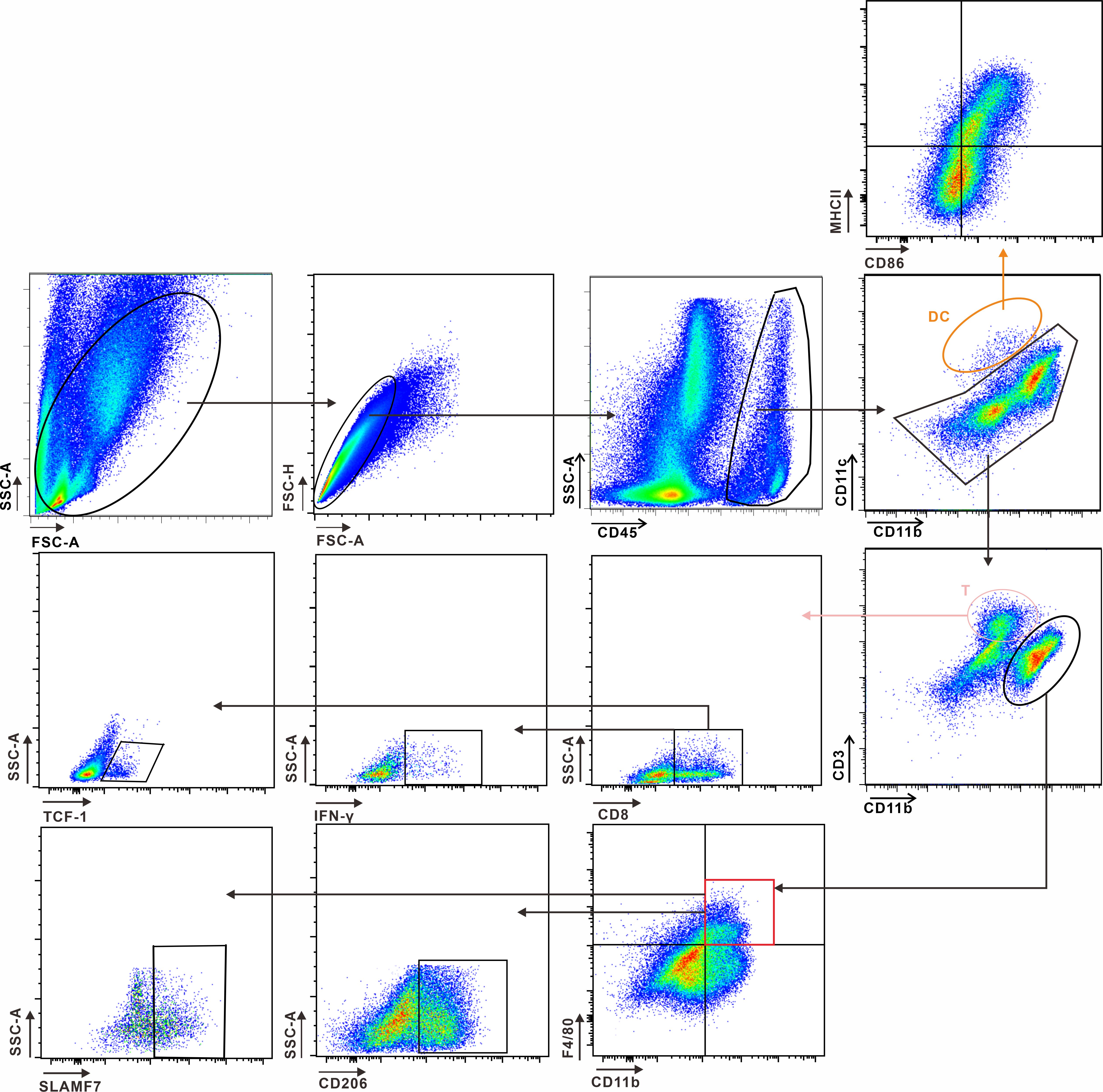


Figure S7.Gatingstrategy to distinguish different immune cell types from the brain sample after given different treatments.


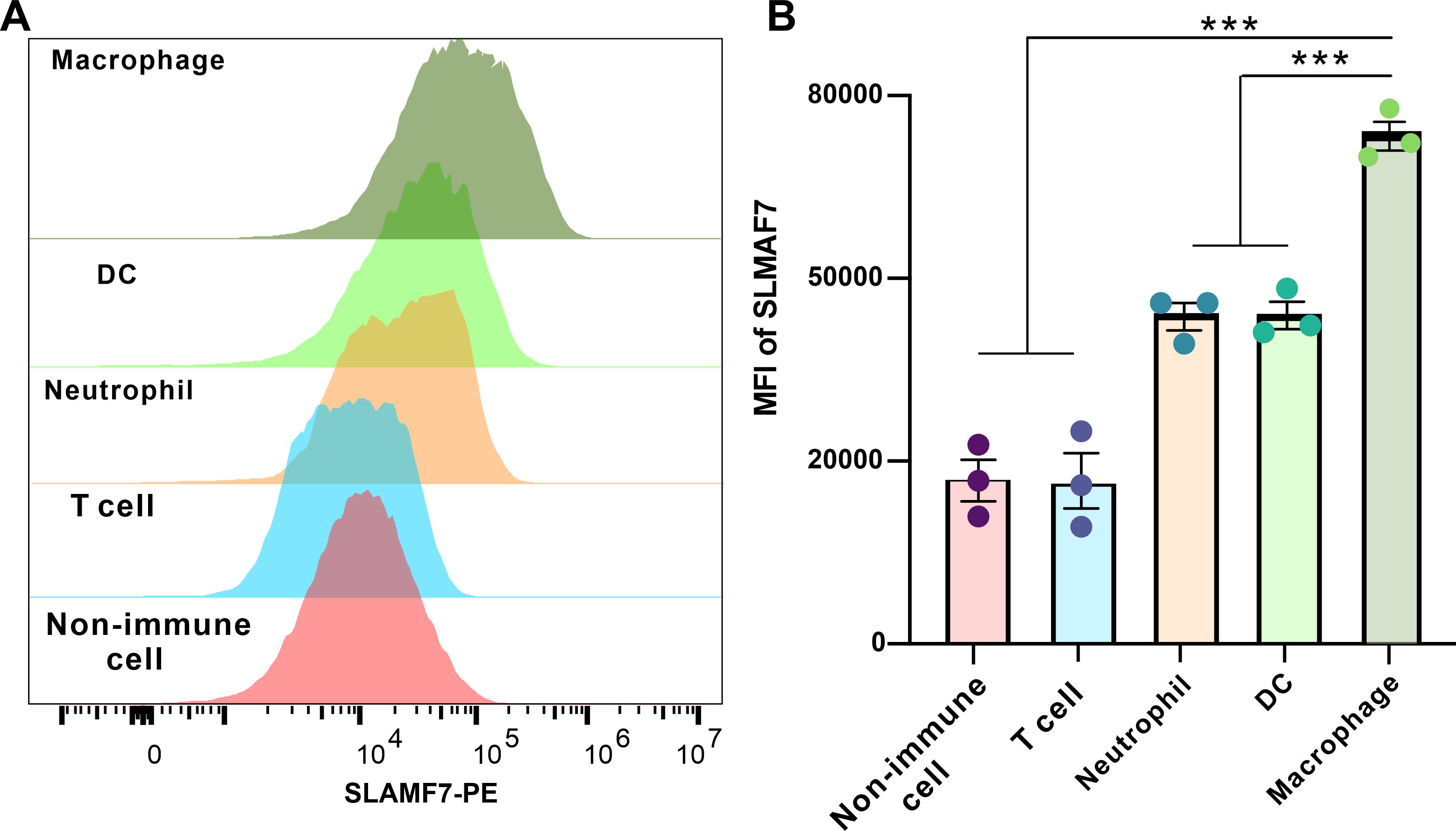


Figure S8.Analysis of SLAMF7 expression on the surface of different immune cells by flow cytometry. Statistical analysis was performed using one-way ANOVA with Tukey's multiple comparison test. Data are presented as the mean ± SD. *P < 0.05, **P < 0.01,***P < 0.001.
